# Quantification and modeling of macroparticle-induced mechanical stress for varying shake flask cultivation conditions

**DOI:** 10.3389/fbioe.2023.1254136

**Published:** 2023-09-04

**Authors:** Marcel Schrader, Kathrin Schrinner, Laura Polomsky, Dimitri Ivanov, Ingo Kampen, Carsten Schilde, Rainer Krull, Arno Kwade

**Affiliations:** ^1^ Institute for Particle Technology, Technische Universität Braunschweig, Braunschweig, Germany; ^2^ Center of Pharmaceutical Engineering, Technische Universität Braunschweig, Braunschweig, Germany; ^3^ Institute of Biochemical Engineering, Technische Universität Braunschweig, Braunschweig, Germany

**Keywords:** macroparticle-enhanced cultivation, CFD-DEM simulation, shake flask, mechanical stress, filamentous microorganism, rebeccamycin

## Abstract

In biotechnological processes, filamentous microorganisms are known for their broad product spectrum and complex cellular morphology. Product formation and cellular morphology are often closely linked, requiring a well-defined level of mechanical stress to achieve high product concentrations. Macroparticles were added to shake flask cultures of the filamentous actinomycete *Lentzea aerocolonigenes* to find these optimal cultivation conditions. However, there is currently no model concept for the dependence of the strength and frequency of the bead-induced stress on the process parameters. Therefore, shake flask simulations were performed for combinations of bead size, bead concentration, bead density and shaking frequency. Contact analysis showed that the highest shear stresses were caused by bead-bottom contacts. Based on this, a newly generated characteristic parameter, the stress area ratio (SAR), was defined, which relates the bead wall shear and normal stresses to the total shear area. Comparison of the SAR with previous cultivation results revealed an optimum pattern for product concentration and mean product-to-biomass related yield coefficient. Thus, this model is a suitable tool for future optimization, comparison and scaling up of shear-sensitive microorganism cultivation. Finally, the simulation results were validated using high-speed recordings of the bead motion on the bottom of the shake flask.

## 1 Introduction

In many process engineering unit operations, mechanical stresses occur in addition to thermal or chemical stresses. Understanding and controlling the positive or negative effects of these stresses is essential to efficiently achieve the desired process objectives. In biotechnology, filamentous microorganisms are particularly sensitive to mechanical stress due to their complex cellular morphology (Nienow, 2020; Böl et al., 2021). The selected bioreactor type and process parameters, such as the specific power input due to agitation and aeration, determine the mechanical stress level (Smith et al., 1990; Jüsten et al., 1996; Lin et al., 2010; Buffo et al., 2016). Depending on the mechanical stress and other cultivation parameters biomass growth in the form of dispersed mycelium, clumps or dense bio-agglomerates (pellets) (Papagianni, 2004; El Enshasy, 2022). The product formation of chemically or pharmaceutically relevant substances is often closely related to the cellular morphology (Braun and Vecht-Lifshitz, 1991; Grimm et al., 2005; Veiter et al., 2018). For the industrially relevant stirred tank bioreactor (STB), the literature contains studies that have investigated and described the influence of mechanical stress on the cellular morphology and productivity at different scales (Li et al., 2002; Casas López et al., 2005; Rocha-Valadez et al., 2007; Böhm et al., 2019; Waldherr et al., 2023). In the work of Reuß (1988), a model for comminution processes in stirred media mills (Weit and Schwedes, 1986) was applied to the disruption of microorganism cells. In this case, the power consumption per flow rate was chosen as the key parameter describing the process. However, this parameter was found to be inapplicable in the studies by Smith et al. (1990). Instead, the concept of the dispersion zone introduced by van Suijdam and Metz (1981) was further developed. Within this dispersion zone, hyphal breakage is possible because the fluid shear stresses are greater than the hyphal cell wall strength. For this, the fracture probability, $p_{f}\propto P\;d_{s}^{-3}t_{c}^{-1}$ , was proposed, where P is the power consumption, $d_{s}$ is the stirrer diameter and $t_{c}$ is the circulation time. In this context, $t_{c}$ , which can also be correlated with the mixing time (Ascanio, 2015), is a measure of the frequency ( $t_{c}^{-1}$ ) for an object (e.g., a bio-pellet) to pass through the shear-intensive dispersion zone. Later, Jüsten et al. (1996) added the influence of stirrer geometries and trailing vortices to the newly defined energy dissipation circulation function (EDCF). This concept has been successfully applied several times, as summarized by Böhm et al. (2019), to correlate variables such as cellular morphology, productivity or growth at different reactor scales and across various filamentous biological systems. Following the EDCF concept, Liu et al. (2016a) used data from computational fluid dynamic (CFD) simulations to correlate the specific death rate with the product of the maximum shear stress and the shear stress frequency. To quantify the hydrodynamic stress in a stirred tank bioreactor (STB) and in a shake flask (ShF), the ratio of the maximum local volumetric power consumption to the mean volumetric power consumption ( $P_{v,\max}{/P}_{V}$ ) was calculated from drop size measurements (Büchs and Zoels, 2001; Peter et al., 2006a; Daub et al., 2014a; Daub et al., 2014b). In the STB, most of the power consumption takes place in a relatively small volume near the stirrer, and only a small amount of power is consumed in the remaining volume. In contrast, the power consumption in a ShF is more uniformly distributed over the wall and bottom, which are relatively large elements. Consequently, $P_{v,\max}{/P}_{V}$ is about one magnitude lower in ShFs ( $P_{v,\max}{/P}_{V}\approx 1-7$ ) than in STBs. This provides a quantitative explanation for the common observation that pellets typically grow to significantly larger sizes in ShF than in STB cultivations (Büchs and Zoels, 2001; Peter et al., 2006a).

One way to increase the mechanical stress in a ShF culture is to add beads. A large number of studies in the literature describe positive or negative effects of additional beads in the lower millimeter range (Hotop et al., 1993; Dobson et al., 2008; Liang et al., 2008; Lee et al., 2010; Sohoni et al., 2012; Cai et al., 2014; Holtmann et al., 2017; Walisko et al., 2017; Song et al., 2018; Zhu et al., 2018; Schrader et al., 2019; Du et al., 2020; Jezkova et al., 2021; Králová et al., 2021; Schrinner et al., 2021; Li et al., 2022). For example, in studies by Dobson et al. (2008) and Lee et al. (2010), an increase in the number of beads enhanced the product formation of geldanamycin and cephalosporin C, respectively, while also affecting the cellular morphology. On the other hand, Cai et al. (2014) observed a decrease in the product formation of the antitumor polyketide aspergiolide A when an increasing number of glass beads ( ⊘= 5 mm) were added to the cultivations of *Aspergillus glaucus*. In another study, an increased number of beads also showed a negative effect on the product formation of the antibiotic natamycin by a filamentous bacterium *Streptomyces gilvosporeus* LK-196 (Liang et al., 2008). Furthermore, Zhu et al. (2021) found the highest increase in concentration of three angucycline/angucyclinone derivatives at a bead size of 500 µm when using glass beads from 100 to 2,000 µm. In the study by Li et al. (2022) macroparticles were also used in cultures of *Aspergilles niger* to enhance the bioleaching of uranium from low-grade ores through the oxalic and citric acids produced. After varying shaker speed, bead size, and number of beads, extraction was enhanced by approximately 10% at a bead size of 0.5 mm and a bead concentration of 40 g L^-1^. In addition, mathematical modeling of the mechanical stress was performed with a slightly modified definition of stress energy (SE) according to the mill model of Kwade (2004). For this purpose, the bead concentration was used in the model instead of the bead density. The bead velocity was estimated from the circumferential velocity at the maximum ShF radius. In addition to ShF, beads were used in microtiter plate cultivations of *Streptomyces tardus* sp. nov (Králová et al., 2021)*,* which produces the antifungal compound candicidin. Also Sohoni et al. (2012) used beads in cultivations of *Streptomyces coelicolor*, which produces the antibiotics undecylprodigiocin and actinorhodin. Varying parameters such as reactor geometry and size, shaking frequency, bead properties (density, number, size) affect the magnitude and frequency of the bead-induced shear stress. Therefore, it is challenging to compare the observed effects of beads without any characteristic quantity like the EDCF for STBs. However, there is no deep systematic understanding of the dependencies of stress magnitude and frequency on these parameters in this application area.

The modeling (Kwade, 2003; Kwade, 2004; Fragnière et al., 2018) and simulation-based quantification (Beinert et al., 2014; Beinert et al., 2018; Trofa et al., 2020; Cabiscol et al., 2021; Fragnière et al., 2021; He et al., 2022) of stresses due to grinding media bead collisions is common in comminution processes. Numerical flow simulations allow a temporally and spatially resolved characterization of bioreactors by solving the mass and momentum balance (Werner et al., 2014). To model multiphase flow in shake flasks, the volume-of-fluid (VOF) method is used to obtain data on mass transport, power consumption, and shear stress (Zhang et al., 2005; Li et al., 2013; Liu et al., 2016b). For example, Liu et al. (2016b) were able to compute a threshold value for the shear stresses on plant cell aggregates at which the specific growth rate decreases. To characterize the bead stresses when macroparticles are used in cultures of the filamentous bacterium *L. aerocolonigenes*, Schrader et al. (2019) combined the CFD simulation approach for shake flasks with the discrete element method (DEM). The DEM, proposed by Cundall and Strack (1979), allows the simulation of particle motion by solving Newton’s second law. In the unresolved CFD-DEM coupling, the momentum exchange between the fluid and the particles is achieved via force models (Bérard et al., 2020). For the analysis of the bead contacts, Schrader et al. (2019) used the evaluation of Beinert et al. (2015) who determined the stress frequency (SF) and stress energy (SE) in mills using CFD-DEM simulations. Previous cultivations of *Lentzea aerocolonigenes* and related simulations were limited to the variation of bead size at constant shaker speed and mass concentration. As a result, other process parameters such as bead number, bead density, bead size, and shaking frequency have been experimentally varied by Schrinner et al. (2021).

Depending on the selected parameters, the concentration of the secondary metabolite rebeccamycin, which has antibacterial and antitumoral properties (Pommerehne et al., 2019), could be significantly increased (Schrinner et al., 2021). In addition to influencing the product titer, morphological and physiological measurements using microscopy and flow cytometry were performed (Schrinner et al., 2020). In this study, the addition of 0.969 mm beads resulted in smaller and rounder pellets, as well as an increase in pellet viability and autofluorescence. In general, the improved rebeccamycin production due to the addition of macroparticles does not seem to be mainly related to the changes in pellet macromorphology (Walisko et al., 2017; Schrinner et al., 2020). A relationship with biomass concentration was also considered unlikely due to the mostly similar bio-dry mass concentrations (Schrinner et al., 2021). Instead, changes in pellet micromorphology, internal pellet structure and mechanically induced stimulation of metabolic activity have been discussed (Schrinner et al., 2020). Furthermore, a potential connection with the antibiotic-forming secondary mycelium investigated by (Manteca and Yagüe, 2018; Manteca et al., 2019) was hypothesized (Schrinner et al., 2021).

In the present study, CFD-DEM simulations were performed in order to determine the dependence of bead-induced mechanical stress for previously experimentally investigated parameter combinations. The numerically results were then combined with cultivation data of filamentous *L. aerocolonigenes* to gain general insights into the relationship between the production of the antibiotic rebeccamycin and mechanical stress. To validate the simulation results, the movement of the beads at the bottom of the ShF was recorded using a high-speed camera. The bead velocities were determined from the high-speed camera images using particle tracking velocimetry (PTV) and compared with the simulations.

## 2 Methods and models

### 2.1 CFD-DEM simulation of the shake flask

The performed multiphase simulations of a 250 mL baffled ShF filled with glass or ceramic beads (Sigmund Lindner GmbH, Warmensteinach, Germany) and 50 mL cultivation medium are based on the CFD-DEM simulation setup established by Schrader et al. (2019). Therefore, only basic information, changes and enhancements in the setup or post-processing are provided in the following sections. For more information on the models and equations used, the reader is referred to the previous work.

#### 2.1.1 Simulation set-up and parameters

The used simulation model is based on the open source software package CFDEMcoupling^®^ (Goniva et al., 2012). This package combines the DEM code LIGGGHTS^®^ (Kloss et al., 2012) and the CFD code OpenFOAM^®^ (version 4) for the simulation of multiphase flows. For LIGGGHTS^®^ and CFDEMcoupling, the academic version published by the department of particulate flow modeling (PFM, JKU Linz, Austria) was used. The transient turbulent flow inside the shake flask was simulated with the volume-of-fluid (VOF) solver “cfdemSolverMultiphase” (Vångö et al., 2018). In short, the VOF approach uses a single momentum and mass balance equation for all phases (Hirt and Nichols, 1981; Brackbill et al., 1992). To track the phase interface, the distribution of the liquid and gas phase fractions is calculated by a transport equation of the phase void fraction (Gueyffier et al., 1999). A Reynolds-averaged Navier-Stokes (RANS) approach was used in combination with a renormalization group (RNG) k-epsilon model for the modeling of the turbulence (Li et al., 2013). The centrifugal forces acting inside a ShF on orbital shaker (Certomat S ll, Sartorius AG, Göttingen, Germany) with an orbital amplitude of 50 mm were considered by imposing a centrifugal acceleration on the fluid phase and the beads. For this purpose, the gravity field of fluid solver was extended in x- and *y*-direction to include the centrifugal acceleration (Schrader et al., 2019). In the unresolved CFD-DEM coupling, the CFD cells are too large to directly calculate the flow between the particles and the resulting forces. For this reason, the momentum exchange between the fluid and solid phases is realized by force models for drag, pressure gradient, surface tension, and viscous forces (Bérard et al., 2020). In detail, the Di Felice model was used for the drag force (Di Felice, 1994). The void fraction field was calculated using the *divided* model approach of the of the CFDEM framework and smoothed using a diffusion based equation (Pirker et al., 2011). On the DEM side, the Hertz-Mindlin model was used to calculate contact forces in the normal and tangential direction (Tsuji et al., 1992; Di Renzo and Di Maio, 2004; Di Renzo and Di Maio, 2005). The constant directional torque (cdt) model was applied as rolling friction model (Ai et al., 2011).

In the previous simulations (Schrader et al., 2019), the ShF geometry had to be slightly extended in both horizontal planes to ensure that the beads were always inside the CFD mesh. As a result, some beads, especially near the curved sidewall, were no longer inside the first boundary cells. Therefore, the open-source meshing tool *cfMesh* (Juretić, 2014) was used to generate a new mesh without artificially increasing the dimensions. This CFD mesh was able to describe the curved flask geometry more accurately while maintaining cell dimensions similar to the first mesh. The velocity boundary condition was set to *noSlip* at the wall of the shaking flask and *pressureInletOutletVelocity* at the opening of the shaking flask. The pressure boundary condition was set to *zeroGradient* at the wall and *totalPressure* at the opening. For a more accurate description of the wetting behavior of the liquid on the shake flask wall, the contact angles listed in Table 1 were used instead of the 90° contact angle used in the past.

**TABLE 1:** Simulation parameters for glass (Sigmund Lindner GmbH, 2017a; Sigmund Lindner GmbH, 2017b; Schrader et al., 2019) and ceramic beads (Beinert et al., 2015; Sigmund Lindner GmbH, 2015), cultivation medium (28°C), water [interpolated for 23°C (Vargaftik et al., 1983; VDI, 2013)] and air [interpolated for 23 or 28°C (VDI, 2013)].

Bead parameter		Glass	Ceramic		Unit
Poisson’s ratio	$\nu_{b}$	0.25	0.31		(−)
Young’s modulus	$Y_{b}$	$6.3∙10^{10}$	$1.0∙10^{11}$		(Pa)
Reduced Young’s modulus	$Y_{b,r}$	$6.3∙10^{6}$	$1.0∙10^{7}$		(Pa)
Density	$\rho_{b}$	2500	3800		(kg m^-3^)
Coefficient of restitution	COR	0.90	0.92		(−)
Friction coefficient	µ	0.30	0.15		(−)
Rolling friction coefficient	$\mu_{r}$	0.10	0.01		(−)

Fluid properties		Liquid	Air		Unit
Medium density	$\rho_{m}$	1001.60 (measured)	1.16		(kg m^-3^)
Water density	$\rho_{w}$	997.54	1.18		(kg m^-3^)
Medium dynamic viscosity	$\eta_{m}$	0.92 (measured)	$1.86∙10^{-2}$		(mPa s)
Water dynamic viscosity	$\eta_{w}$	0.93	$1.84∙10^{-2}$		(mPa s)
Medium surface tension	$\sigma_{m}$	57.05 (measured)			(mN m^-1^)
Water surface tension	$\sigma_{w}$	72.28			(mN m^-1^)
Medium contact angle	$\theta_{m}$	32.79 (measured)			(°)
Water contact angle	$\theta_{w}$	49.50			(°)

		DEM		CFD	Unit
Time step	∆t	$1.0∙10^{-6}$		$1.0∙10^{-5}$	(s)
CFD-DEM coupling interval	c	10			(−)

Previously, the hydrodynamic interactions between an approaching and separating pair of beads near the contact were considered by applying a lubrication force model. To account for sliding forces and moments as well as torsional moments in addition to normal forces, the corresponding model equations of Kroupa et al. (2016) were implemented in the DEM code. Due to the divergence of the lubrication forces and moments at an infinitesimally small separation distance, a limit was set at a separation distance (slip length) of 10% of the bead radius. Additional simulation parameters are shown in Table 1.

The coefficients of friction for particle-particle and particle-wall contacts were assumed to be identical because the wall and beads are made of glass. Ceramic and glass contacts were also assumed to have the same friction coefficient as ceramic-ceramic contacts. In addition to the fluid properties for the cultivation medium, the values for water are given, because water was used for validation.

#### 2.1.2 Simulation parameter combinations

Based on the macroparticle-enhanced cultivations of Schrader et al. (2019) and Schrinner et al. (2021) several simulation series (see Table 2) were performed with different combinations of shaking frequency f , bead diameter $d_{b}$ , bead density $\rho_{b}$ and bead volume concentration $c_{v,b}$ . In the first series, different bead sizes (0.540, 0.658, 0.969, 1.183, 1.513, 1.746, and 1.932 mm) were tested at different shaking frequencies and constant bead concentration. Since the number of beads $N_{b}$ changes when the bead concentration is constant and the bead diameter varies, different bead sizes and a constant number of 4,200 beads were used in another study. Glass and ceramic bead mass concentrations were varied to study the effect of bead number at constant bead size. To improve the comparability of the bead concentrations, the bead volume concentrations are also given in Table 2.

**TABLE 2:** Overview about all parameter combinations used in the simulations.

Variation of	Shaking frequency	Bead diameter	Bead density	Bead volume concentration
	f	$d_{b}$	$\rho_{b}$	$c_{v,b}$
	(min^-1^)	(mm)	(kg m^-3^)	(mL L^-1^)
Bead diameter $d_{b}$ for different f	100–200	0.540–1.932	2,500	40.0
Bead diameter $d_{b}$ ( $N_{b}=4200$ )	120	0.540–1.932	2,500	6.9–317.2
Glass bead number $N_{b}$	120	0.969	2,500	10.0–60.0
Ceramic bead number $N_{b}$	120	0.918	3,800	6.6–59.2

### 2.2 Simulation post-processing

#### 2.2.1 Stress energy and frequency and bead velocity

The stress energy (SE) according to the analytical model for stirred media mills (Kwade, 2003) is defined as

$$SE\propto SE_{b}=d_{b}^{3}∙v_{t}^{2}∙\rho_{b}$$ (1)

In this study, the maximum achievable SEs for different contact types were calculated analogously to the energy equations described by Beinert et al. (2015). A distinction is made between normal, shear, torsional and rolling bead-bead or bead-wall contacts. Depending on the type of contact, the corresponding kinetic energy is calculated using the translational or rotational relative velocity instead of the stirrer tip velocity $v_{t}$ . These kinetic energies correspond to the maximum possible SE in the cultivation process. The SF is defined as the number of bead-bead or bead-wall contacts per second. Instead of a constant time interval for the evaluation of the bead contacts, a fixed number of six shaker rotations was chosen. This avoids potential over- or under-evaluation of the temporally fluctuating SE when the shaking frequency is changed. Within the six shaker rotations, all new bead contacts that occurred in a time step were saved. A cumulative distribution of the SE was then calculated from the list of all contacts of for six rotations. Therefore, the distribution is not a time average. Instead, it represents the SE spectrum within the time period under consideration. The translational shear velocity of the beads was calculated from the translational shear energy $E_{ts}$ based on Eq. 2 (Beinert et al., 2015).

$$E_{ts}={\frac{1}{2}∙\frac{\pi }{6}∙d}_{b}^{3}∙v_{b,ts}^{2}∙\rho_{b}$$ (2)

The translational bead velocities, used in the validation (see chapter 3.5), were read out at 0.01 s intervals for a period of five shaker rotations and saved in a list. This list was then used to calculate a cumulative distribution of bead velocity based on the evaluated DEM data for five shaker rotations.

#### 2.2.2 Volumetric power consumption

The mean volumetric power consumption $P_{v}$ in the cultivation medium, which also depends on the shaking frequency, was calculated from the turbulent energy dissipation rate ϵ and the medium density $\rho_{m}$ according to the following equation (Li et al., 2013):

$$P_{v}=\epsilon ∙\rho_{m}$$ (3)

Data were analyzed every 0.01 s to calculate the time averaged power consumption for the last five shaker rotations.

### 2.3 Validation of the bead motion via particle tracking velocimetry (PTV)

Particle tracking velocimetry (PTV) was applied for the validation of the three-phase simulations of the ShF. In contrast to particle imaging velocimetry (PIV), a Lagrangian frame of reference is used to determine the velocity of a bead from its displacement during the elapsed time between two recorded images (Dracos, 1996). For this purpose, an experimental setup and subsequent image processing were established in this work in order to determine bead velocities.

#### 2.3.1 Experimental set-up to record bead and liquid motion

To track the complex motion of the liquid and the beads from below the ShF, an experimental setup was constructed (Supplementary Figure S1). An aluminum plate with four vertical threaded rods was screwed onto the base plate of the orbital shaker. Afterwards, a 250 mL ShF with four baffles, already filled with 50 mL of water and beads, was clamped between two transparent polymer plates at a height of about 40 cm. To increase the stability of the experimental setup, two 3D-printed brackets and guy ropes (for 160 min^−1^ only) were installed. A high-speed camera (Promon 501, AOS Technologies AG, Switzerland) mounted vertically below the center of the ShF recorded images (896 × 920 pixels) at a frame rate of 200 s^−1^. Optimal illumination of the ShF was crucial to minimize disturbing image artifacts caused by light refraction and reflection on the water surface. On the one hand, a square 30 cm LED panel was placed statically above the orbital shaker. On the other hand, a circular LED strip was installed at the level of the bottom of the ShF. A cardboard cylinder, covered on the inside with aluminum foil, was used for homogeneous illumination. Glass (1.183, 1.513, and 1.932 mm) and ceramic (0.918 mm) beads (Sigmund Lindner GmbH, Warmensteinach, Germany) were used in the experiments at shaking frequencies of 100, 120, and 160 min^−1^. In contrast to the simulations, the minimum bead size was limited to approximately 1 mm, as smaller beads could not be reliably detected in the image analysis. The transparent glass beads were difficult to identify in the images during pre-processing. Therefore, a mixture of 26% black coated marker beads and 74% transparent beads of the same size was chosen. In the case of white ceramic beads, it was not possible to use beads of a different color, so the amount of beads was reduced from 40 mL L^−1^ (glass) to 2.63 mL L^−1^ to avoid overlapping beads in the images.

#### 2.3.2 Calculation of the particle velocity distribution

Prior to evaluation, the images were pre-processed using MATLAB^®^ to separate the beads from the background structures of the liquid-gas phase interface. In the case of glass beads, the imported images were converted to grayscale and smoothed with a flat-field correction. In the second step, a binary image was generated using an adaptive threshold. Then, the centers of circular objects within a defined radius range were detected in order to draw a new image with only the detected beads as white circles on a black background. In the case of white ceramic beads, an adapted image pre-processing was necessary, since no black marker beads could be used. In addition to image optimization and filtering steps, a marker-controlled watershed separation was applied to separate the partially overlapping beads. In addition, the centers of the beads were mapped to generate a new image with non-overlapping circles for PTV analysis. The actual calculation of bead velocities out of the pre-processed images was performed using the open-source MATLAB^®^ tool *PTVlab* (version 1.0.0.0) (Brevis et al., 2011; Patalano and Wernher, 2013). The number of images used to calculate the velocity distribution was the equivalent number of images taken during five rotations of the shaker. Therefore, the distribution is not a time average. Instead, it represents the velocity spectrum within the time period under consideration. In detail, the *Gaussian mask* option was selected for particle detection and the cross-correlation method for particle tracking. By default, *PTVlab* provides an interpolated velocity field whose expansion is determined by the spatial distribution of the beads. As a result, bead velocities that do not exist were assumed in zones without beads. For this reason, the *PTVlab* source code was extended to output the discrete velocity distribution without spatial interpolation.

## 3 Results and discussion

After the analysis of the contact types (section 3.1), the effect of different experimental parameters on the bead induced stress is considered (sections 3.2 and 3.3). For this purpose, simulations were performed and evaluated for different shaking frequencies, bead numbers, bead diameters and bead densities. The parameter sets used are mainly based on macroparticle-supplemented cultivations of the filamentous bacterium *L. aerocolonigenes* (Schrader et al., 2019; Schrinner et al., 2021). In addition to the evaluation of the bead collisions, the volumetric power input (section 3.4) was determined by simulation for different shaking frequencies and bead sizes. In section 3.5 the translational bead motion on the ShF bottom is evaluated using high-speed camera images to validate the simulations. Finally, in section 3.7, the product concentrations and the average yield coefficients are linked to the newly derived characteristic parameter from section 3.6.

### 3.1 Stress energy distribution and fraction of contact types

**FIGURE 1:**
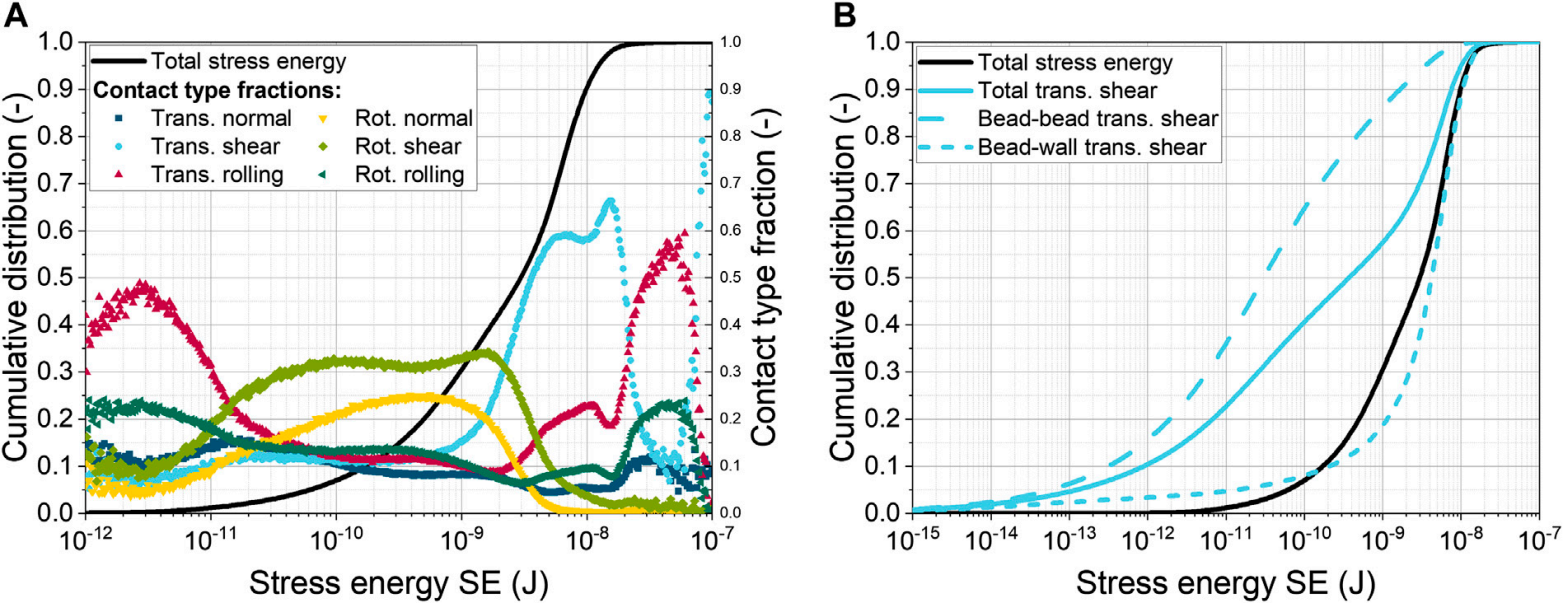
**(A)** Cumulative distribution of total stress energy (SE) for 0.969 mm glass beads at 120 min^−1^ with 40 mL L^−1^ and fractions of six contact types; **(B)** Cumulative distribution of total and translational shear SE (bead-bead and bead-wall contacts).

The total SE represents the sum of the SEs for six different contact types, that rely on impact, shear, rolling, and torsion. Figure 1A shows the sum distribution of the total SE as well as the fractions of each contact type.

Compared to the first shake flask simulation by Schrader et al. (2019) of the identical setup (120 min^−1^, 0.969 mm glass beads), a slight shift of the sum distribution towards lower energies can be observed (data not shown). In addition, the saddle point in the sum distribution is missing in the current simulation. Another difference is that the order of the contact type fractions has shifted. Previously, translational normal and shear contacts dominated in the lower energy range, while the maximum SEs were caused by translational shear to almost 90%. In the latest results, the rotational shear and normal contacts dominate in the lower energy range with about 30% and 25%, respectively. All other contact types have only a small share of 10% of the total SE. The highest SE is still caused by translational shear with a fraction of about 60%. The reasons for the observed differences are, on the one hand, the more precise shape description of the SF geometry by the CFD mesh and, on the other hand, the application of a new lubrication force model from Kroupa et al. (2016). In detail, the boundaries of the CFD and DEM simulation domains are closer together in the new mesh, resulting in a more accurate calculation of the coupling forces near the wall is achieved. Moreover, the new lubrication force model includes additional calculations of tangential forces, as well as sliding and twisting moments, which in together influence particle motion.

Figure 1B shows not only the total SE, but also the total SE for translational shear. This distribution is formed by the sum of the cumulative SE distributions for bead-bead and bead-wall translational shear contacts (dashed lines). It can be seen that the translational shear SE between the beads is lower than that between the beads and the wall of the ShF. Furthermore, the distribution for wall contacts in the upper third is close to the total SE distribution, indicating that this type of contact is decisive for the maximum bead SE. This conclusion confirms the common assumption in the literature that the addition of beads increases the shear stress on filamentous microorganisms (Dobson et al., 2008; Liang et al., 2008; Song et al., 2018).

In cultivation, shear forces can break bio-agglomerates such as pellets or even suppress their formation. In addition, individual exposed hyphae can be sheared off the pellet surface (erosion). For this reason, the velocity $v_{b,ts}$ , which is used in the calculation of the bead-wall translational shear SE, is considered in the following sections. This step allows a more differentiated consideration of the influencing factors on the bead induced shear stress. Only a small but unknown fraction of all contacts have velocities high enough to provide effective stress for hyphal breakage. However, in this study, the 90% value of the cumulative SE distribution was used to calculate $v_{90,ts}$ . Nevertheless, excessive normal forces can also cause individual cells to burst (Taubert et al., 2000; Günther et al., 2016; Overbeck et al., 2017). Furthermore, Dittmann et al. (2019), suggested that pellet compression may alter the internal pellet structure due to plastic deformation or cell adhesion effects. Another potential mechanism could be an increase in mass transport due to the squeezing of culture medium out of the pellet.

### 3.2 Dependence of shear bead velocity on cultivation process parameters

#### 3.2.1 Effect of shaking frequency on shear velocity

**FIGURE 2:**
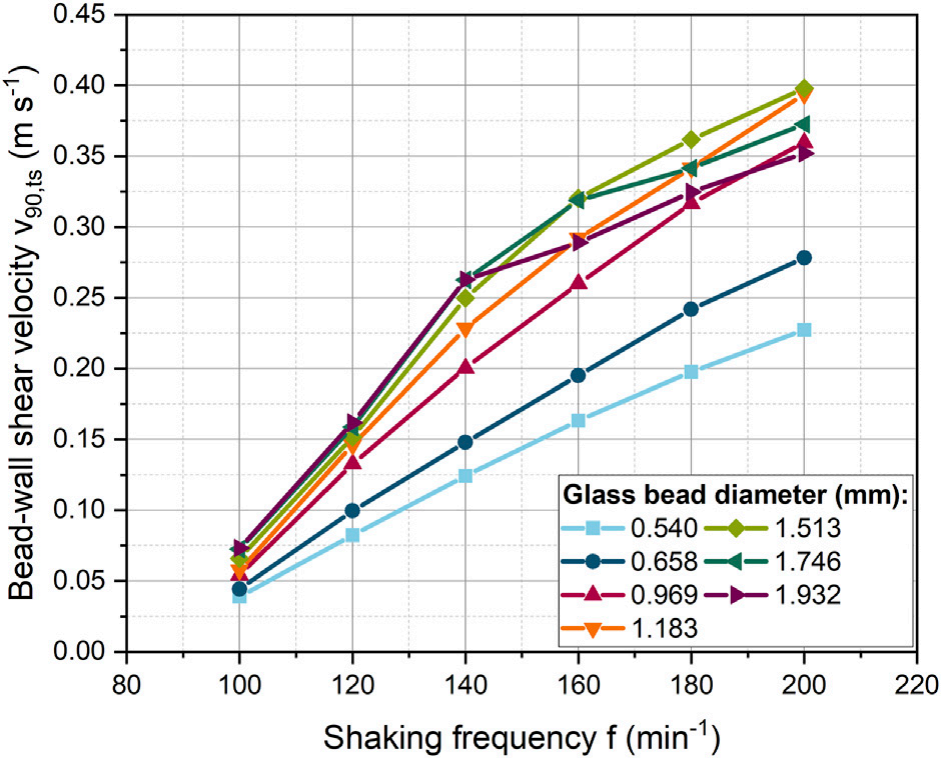
Dependency of the bead-wall shear velocity from the shaking frequency for different glass bead diameters with a constant bead concentration (40 mL L^−1^).

Shaking frequency is the driving force for the beads movement. The bead-wall shear velocity for all bead diameters increases steadily with increasing shaking frequency (Figure 2). It is noticeable that the shear velocity initially increases gradually more strongly up to a shaking frequency of 140 min^−1^ and then the slope decreases. Overall, larger beads reach a higher velocities than smaller ones. However, this is not true for the two largest bead diameters. In the high shaking frequency range, the shear velocities were slightly below the values for a bead size of 1.513 mm.

Forces affecting the bead motion pattern must be considered to explain the observed trends in ShF. For simplicity, the beads are assumed to move with velocity $v_{b}$ on a circular path with radius $r_{rot}$ (Eq. 4). This radius is a function of the shaking frequency, because the centrifugal forces $F_{a}$ , caused by the centrifugal acceleration $a_{c}$ , push the beads outward (Eq. 5). The ShF radius limits the theoretical maximum rotation radius. However, gravity counteracts the outward movement of the bead as soon as a bead enters the curved area of the ShF bottom. This balance of forces, which is also influenced by frictional, fluid-particle interaction and particle contact forces, determines the maximum bead rotation radius. If the centrifugal influence is considered in Eq. 4, this results in a theoretical increase in bead velocity with the cube of the shaker frequency in Eq. 6. Besides, larger beads experience higher centrifugal forces, which can additionally affect the bead motion. Overall, this explains the initial large increase in velocity at the lower shaking frequencies in Figure 2.

$$v_{b}=r_{rot}\left(F_{a_{c}}\right)\cdot 2\pi \cdot f$$ (4)

$$r_{rot}\propto F_{a}\propto a_{c}\cdot m_{b}\propto r_{orb}\cdot f^{2}\cdot m_{b}$$ (5)

$$v_{b}\propto r_{orb}\cdot f^{3}\cdot m_{b}$$ (6)

As the shaking frequency increases, the centrifugal effect gradually decreases because most of the beads are centrifuged to the outer zone of the ShF. As a result, it follows that the bead velocity increases linearly with the shaking frequency.

#### 3.2.2 Effect of bead diameter on shear velocity

**FIGURE 3:**
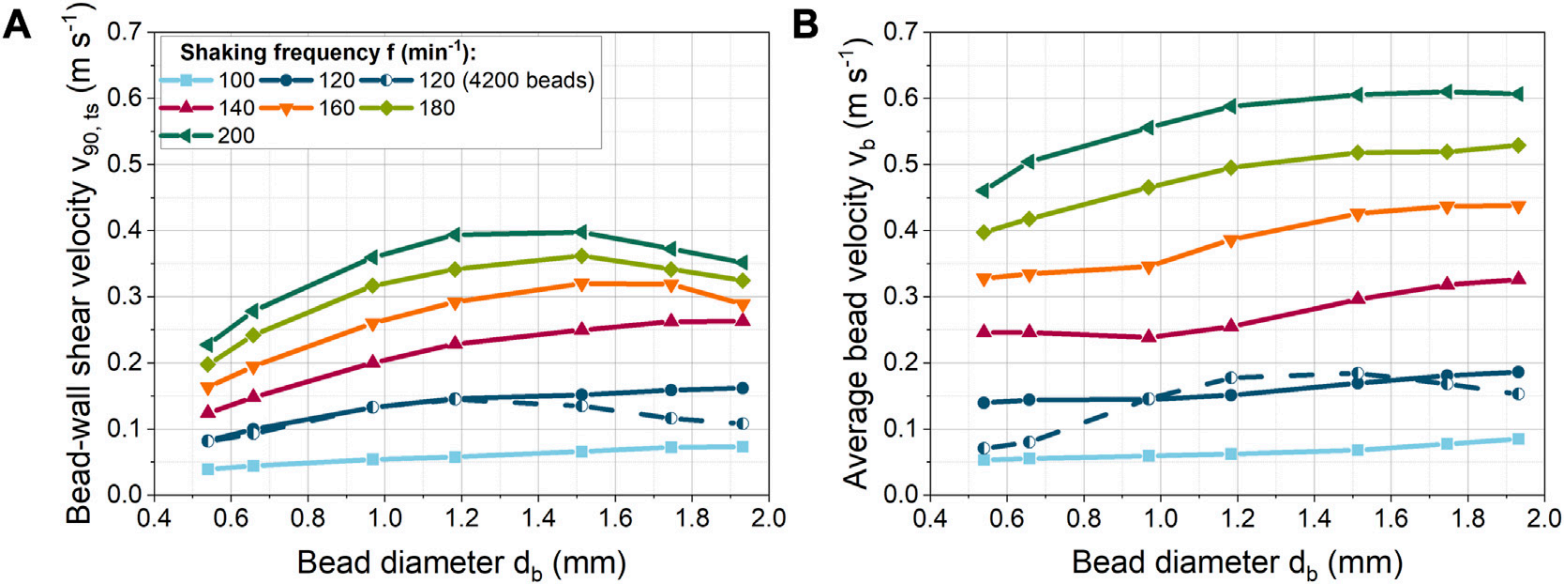
**(A)** Correlation of bead-wall shear velocity with the bead size at a constant volume concentration (40 mL L^−1^, 529–24,257 beads) for different shaking frequencies. In addition at 120 min^−1^, a constant bead number of 4,200 was used; **(B)** Spatially averaged bead velocity ( $v_{b}$ ) in x- and *y*-direction from DEM simulations.

In Figure 3A, the bead diameter was varied from 0.540 to 1.932 mm at a constant bead volume concentration of 40 mL L^−1^ to determine the dependence of bead-wall shear velocity on the bead diameter. For the shaking frequency of 100 min^−1^, only a small linear increase in velocity is observed. Whereas at higher shaking frequencies, the shear velocity increases progressively more strongly up to a bead size of 1.183 mm. For 120 min^−1^ and 140 min^−1^, the shear velocity starts to increase only slightly from a bead diameter of 1.183 mm, while a decrease occurs at even higher shaking frequencies and larger bead diameters. The increase in velocity can be related to the influence of centrifugal forces, which depend on bead mass and shaking frequency (see Eq. 4). In contrast, other bead size dependent forces (e.g., drag, pressure gradient, lubrication, friction forces) increase with increasing diameter. As a result, additional forces dampen the increase in shear velocity with the bead diameter.

Furthermore, for a given bead volume concentration, the number of beads increases with decreasing diameter. As a result, more beads are distributed on the bottom of the flask and new layers of beads are formed on top of the bottom layer (Supplementary Figure S3 and Supplementary Video S3). Consequently, more bead-bead and bead-baffle interactions make a free bead motion more difficult. In addition, new shear planes are created between the bead layers, so that the shear velocity between the beads increases as the number of beads increases (Supplementary Figure S2A).

At higher shaking frequencies, the beads are forced intensively against the curved bottom of the ShF. On the one hand, the beads largely accumulate in layers with increasing rotational speed, resulting in an increased shear velocity between the beads (Supplementary Figure S2A). On the other hand, the normal forces and thus the friction forces on the beads increase. As a result, the beads begin to roll faster instead of sliding over the glass surface of the ShF. This reduces the translational shear velocity portion and conversely increases the translational rolling velocity portion. To illustrate the effect, the average velocity of the beads is shown in Figure 3B. The spatially averaged bead velocity was taken directly from the x- and y-velocity components of the DEM simulations. For example, for 200 min^−1^ it can be seen that the spatially averaged bead velocity stagnates from a bead size of 1.2 mm. In contrast, the shear velocity decreases (Figure 3A). Consequently, if the spatially averaged bead velocity is constant, the rotational velocity of the beads must be increasing. The rotational acceleration of a sphere is inversely proportional to its rotational inertia and rolling friction, according to the equation of rotational motion. Due to the proportionality of the rotational inertia to the mass and size of a bead, larger beads change their rotational speed more slowly than smaller or lighter beads because of the applied moments. Furthermore, tangential forces on a bead cause moments that accelerate or decelerate the rotation depending on the direction of the tangential force. The tangential forces depend on the normal bead forces and the coefficient of friction between the beads. In addition, beads in layers hamper each other’s rotation. In sum, the intensity of translational shear is determined by the rolling behavior of a bead. The complex interplay of influencing factors illustrates that a direct prediction of shear stress by beads is extremely complex or even impossible without simulations.

When comparing the absolute velocities (Figure 3B), it is noticeable that the bead velocities are higher than the shear velocities. Therefore, it should be noted that rolling of the beads may be hindered by the biomass in the cultivation, so that in reality deviating shear velocities can occur.

**FIGURE 4:**
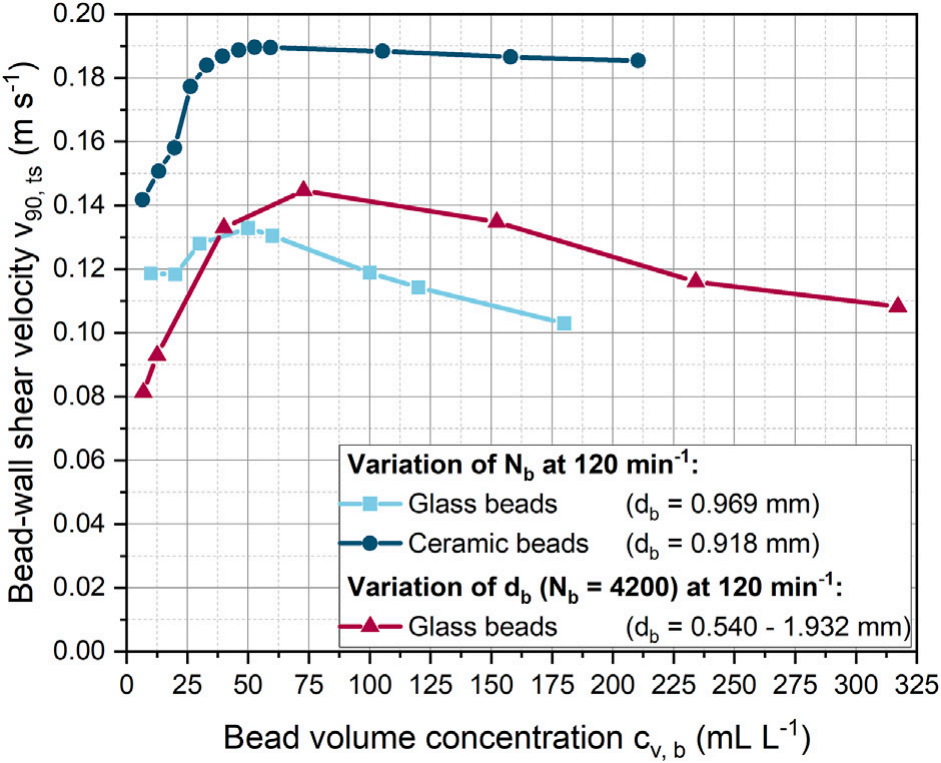
Influence of the glass or ceramic bead volume concentration on the bead-wall shear velocity at varying bead diameter and constant shaking frequency of 120 min^−1^.

In addition to this effect, in Figure 3A, at a shaking frequency of 120 min^−1^ and a constant bead number the shear velocity decreases above 1.183 mm. In this case, the underlying cause cannot only be increased bead rolling, as the average bead velocity also decreases in Figure 3B. In all other simulations with constant bead mass, the number of beads decreased with increasing diameter, so that the reduced number of beads compensated for the increased space requirement per bead. Instead, with a constant number of beads, the ShF bottom fills up more at large bead diameters, making bead motion more difficult. As a result, the velocity of the beads in Figure 4 decreases above a volume concentration of approximately 73 mL L^−1^.

#### 3.2.3 Effect of bead volume concentration on shear velocity

In addition to shaking frequency and bead size, the bead volume concentration was varied at a constant shaking frequency of 120 min^−1^ (Figure 4). For a constant glass bead size of 0.969 mm, the bead-wall shear velocity first increases slightly from 0.12 to almost 0.13 m s^−1^ and then decreases with increasing bead volume concentration. However, in contrast to glass beads, an increase in the number of ceramic beads led to a significantly higher increase in shear velocity. Accordingly, at a bead volume concentration of around 40 mL L^−1^, approximately 43% higher shear velocities were achieved between the beads and the wall of the ShF. Interestingly, the velocity of the ceramic beads decreased only slightly with increasing concentration.

The simulations illustrate (Supplementary Figures S4, S5 and Supplementary Videos S4, S5) that ceramic and glass beads formed layers of beads with increasing bead concentration. Compared to the ceramic beads, the glass beads spread over a larger area of the ShF bottom than the ceramic beads. The higher velocities and greater proportion of bead layers for the ceramic beads can be explained by their 52% higher density, which results in increased centrifugal forces. In addition, the coefficients of friction and rolling friction for ceramics are lower than for glass beads, which in turn can lead to a variation in the rolling and shearing velocity proportions.

### 3.3 Stress frequency as a function of bead number and shaking frequency

**FIGURE 5:**
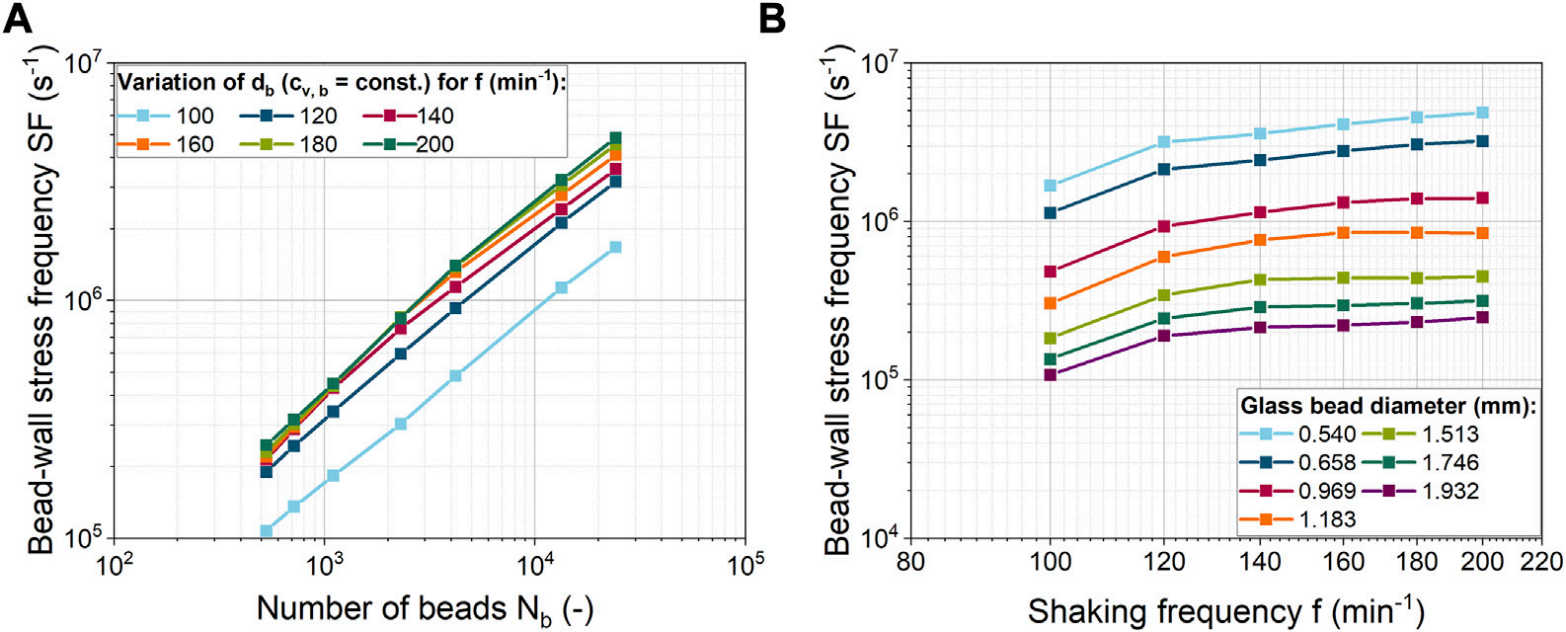
**(A)** Bead-wall stress frequency (SF) for different numbers of glass beads and shaking frequencies; **(B)** Correlation of the bead-wall SF with shaking frequency for different glass bead diameters. All simulations were done at a constant bead volume concentration.

In addition to the shear velocity as a measure of the strength of the mechanical stress, the SF of the mechanical stress is also important. In the EDCF concept for fluid mechanical stress on filamentous microorganisms, the circulation time is used as a measure of the breakup frequency. In contrast, when macroparticles are used in a ShF, the frequency of bead-wall contacts is considered in Figure 5. Due to the constant total bead volume concentration, the SF increases with an exponent in the range of 0.72–0.78 with increasing bead number at decreasing glass bead size (Figure 5A). In the study by Schrinner et al. (2021), the SF was assumed to be directly proportional (exponent of 1) to the number of beads and the shaking frequency. This deviation, which is related to the number of beads, is due to the fact that not all beads are always in contact with the ShF bottom (see Supplementary Figure S3 and Supplementary Video S3). In Figure 5B the SF increases almost linearly for higher shaking frequencies and small bead sizes. The influence of the shaking frequency decreases for larger beads in combination with a lower number of beads.

**FIGURE 6:**
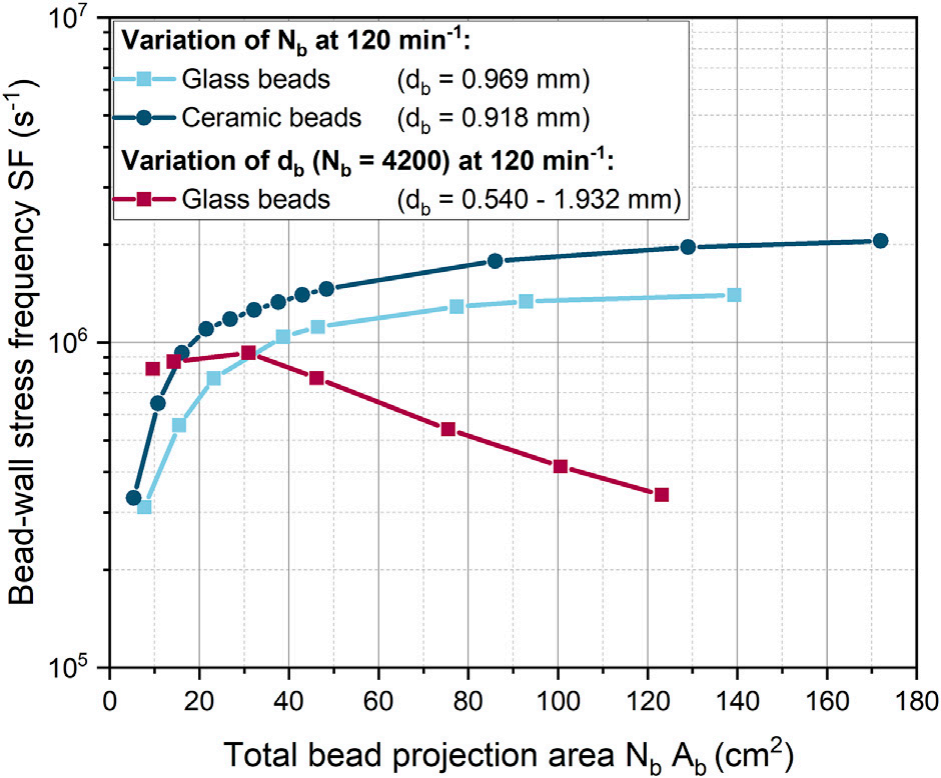
Dependency of the bead-wall stress frequency (SF) on the total projected bead area (which corresponds to the bead volume concentration) for 0.918 mm ceramic beads and glass beads of different diameters at a constant shaking frequency of 120 min^-1^.

In the previous simulations (Figure 5), both the number of beads and the diameter of the beads were varied simultaneously. Instead, in Figure 6, either the number of beads or the size of the beads was varied at a constant shaking frequency. The product of the number of beads $N_{b}$ and the bead cross-projection area $A_{b}$ is the total bead projection area and was considered as a measure of space required by a given number of beads.

For both glass and ceramic beads, the increase in SF is initially pronounced and then gradually decreases. However, the SF increases more rapidly for ceramic beads than for glass beads. The initial rapid increase in SF can be explained by the fact that a portion of the ShF bottom is initially covered with beads. At higher bead concentrations, multiple layers of beads form on the ShF bottom, so that only a reduced number of beads can form new wall contacts on the side walls. The planar surface of the ShF bottom has an area of approximately 25 cm^2^. For a similar total projection bead area, the SF begins to rise more slowly, meaning that from this point on, new contacts can only be added in the curved area. In addition, the bead-bead contacts increase in the upper bead layers, creating a new shear zone. From this point on, the shear velocity between the glass and ceramic beads suddenly increases so that the ratio of bead-wall and bead-bead shear velocities drops sharply (Supplementary Figure S2). As the bead concentration increases, the velocity ratio remains constant, but the shear stress at the wall is always at least two times higher. With regard to the mechanical stress on the microorganisms, it can be assumed that the formation of several layers of beads is rather negative, since the exchange of biomass between beads and bottom is made more difficult. This assumption is confirmed by the fact that in the cultivations of Schrinner et al. (2021) with the same bead parameters, the product formation is at its optimum at a glass bead volume concentration of 40 mL L^-1^. This concentration corresponds to a total projected bead area of about 30 cm^2^, at which the SF begins to increase more slowly. With the ceramic beads, the highest product titer was obtained at a bead volume concentration of 13.2 mL L^-1^ with a total projected bead area of approximately 11 cm^2^ (Schrinner et al., 2021). This is consistent with the observation that SF increases more rapidly with ceramic beads. However, compared to glass beads, the optimum in cultivation occurs at a slightly lower SF.

For 4,200 beads of different sizes, SF remains nearly constant up to a total projected bead area of about 30 cm^2^ ( $d_{b}=0.969\;mm)$ and then decreases continuously. The cross-sectional area of the beads, which increase quadratically with the bead diameter, determines the area required per bead on the ShF bottom. Accordingly, the bottom is more easily covered with larger beads, so that as the bead diameter increases, fewer beads can come into contact with the ShF wall. Similar to the previous variation of the number of beads, the shear velocity between the beads increases when a new bead layer is formed at a bead diameter of 0.969 mm (Supplementary Figure S2).

### 3.4 Mean volumetric power consumption

From a biochemical engineering point of view, the mean volumetric power consumption ( $P_{V}$ ) plays an important role in cultivations, as it correlates in some way with mixing time, oxygen transfer and hydrodynamic stress. For a holistic evaluation of cultivation conditions from Schrinner et al. (2021), $P_{V}$ was determined from the turbulent energy dissipation rate (Eq. 3) for an extended shaking frequency up to 200 min^-1^.

**FIGURE 7:**
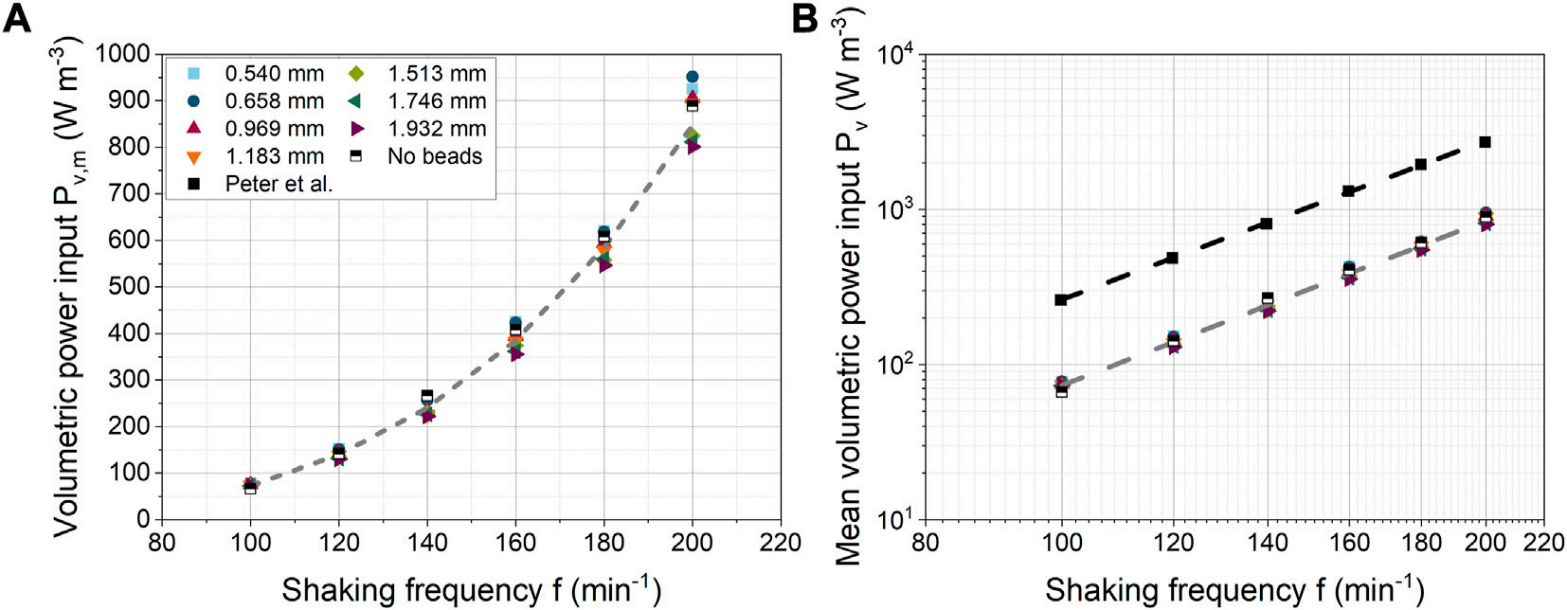
**(A)** Influence of shaking frequency on mean volumetric power consumption for different bead diameters (constant bead volume concentration of 40 mL L^-1^); **(B)** Comparison with experimental data from Peter et al. (2006b).

In Figure 7A, $P_{V}$ increases sharply from about 75 W m^−3^ at 100 min^−1^ to about 900 W m^−3^ at 200 min^−1^. Above 100 min^−1^, $P_{V}$ decreases by 12%–16% with increasing bead diameter, depending on the shaking frequency. Larger beads are expected to reduce the motion of the fluid so that less energy is dissipated by turbulence. Simulations without beads resulted in power inputs in a similar range to those with beads. Thus, the addition of beads appears to have little effect on the turbulent energy dissipation in the fluid. However, only the energy dissipation in the fluid was considered here, so the energy dissipation due to particle-particle and particle-wall interactions is missing.

An exponential fit over all bead diameters gave a mean exponent of 3.53 ± 0.03. In the literature, both experimentally and numerically derived values for power consumption have been reported, although different shaking conditions were used. In the case of a 250 mL unbaffled ShF filled with 25 mL and an orbital diameter of 60 mm, Zhang et al. (2005) determined a correlation of $\epsilon \propto f^{2.7}$ using CFD-simulations. On the other hand, the Büchs group developed a measurement technique to capture the torque for multiple unbaffled ShFs on the orbital shaker machine (Büchs et al., 2000a). For a wide range of shaking conditions, a denpendence of $P_{V}\;∼\;f^{2.8}$ was found. The method allowed the development of a model that introduced a modified power number (Ne’) as a function of the Reynolds number (Re). This correlation specifies how different cultivation parameters influence $P_{V}$ in ShFs (Büchs et al., 2000a). The equation was extended to liquids of higher viscosities in unbaffled ShFs (Büchs et al., 2000b). Later, Peter et al. (2006b) investigated the power consumption of a single baffled ShF with a newly developed device. The results of this study indicated an approximately one order of magnitude higher $P_{V}$ compared to an unbaffled ShF. The reason for the higher power consumption is the disturbance of the fluid movement by the baffles. For comparison with the results of Peter et al. (2006b), $P_{V}$ is shown in Figure 7B as an example for a 300 mL ShF filled with 48 mL deionized water at an identical shaking diameter of 50 mm. While Peter et al. (2006b) measured a higher $P_{V}$ in absolute terms, the dependence on the shaking frequency of 3.4 is close to the value of this study. Apart from the slightly larger flask volume (300 vs. 250 mL) and the sligthly smaller liquid volume (48 vs. 50 mL), the main reason for the absolute higher $P_{V}$ is most likely the depth of the baffles (14 vs approx. 7 mm) and the number of baffles (3 vs. 4). Furthermore, the energy dissipation rate from the turbulence model was used to calculate $P_{V}$ in this study. Due to the limitations of the minimum CFD cell size in unresolved CFD-DEM simulations, additional deviations may occur because the cell sizes may not be small enough to correctly describe the turbulence.

### 3.5 Validation of the shake flask simulations via particle tracking velocimetry

#### 3.5.1 Spatial distribution of the beads on the shake flask bottom

**FIGURE 8:**
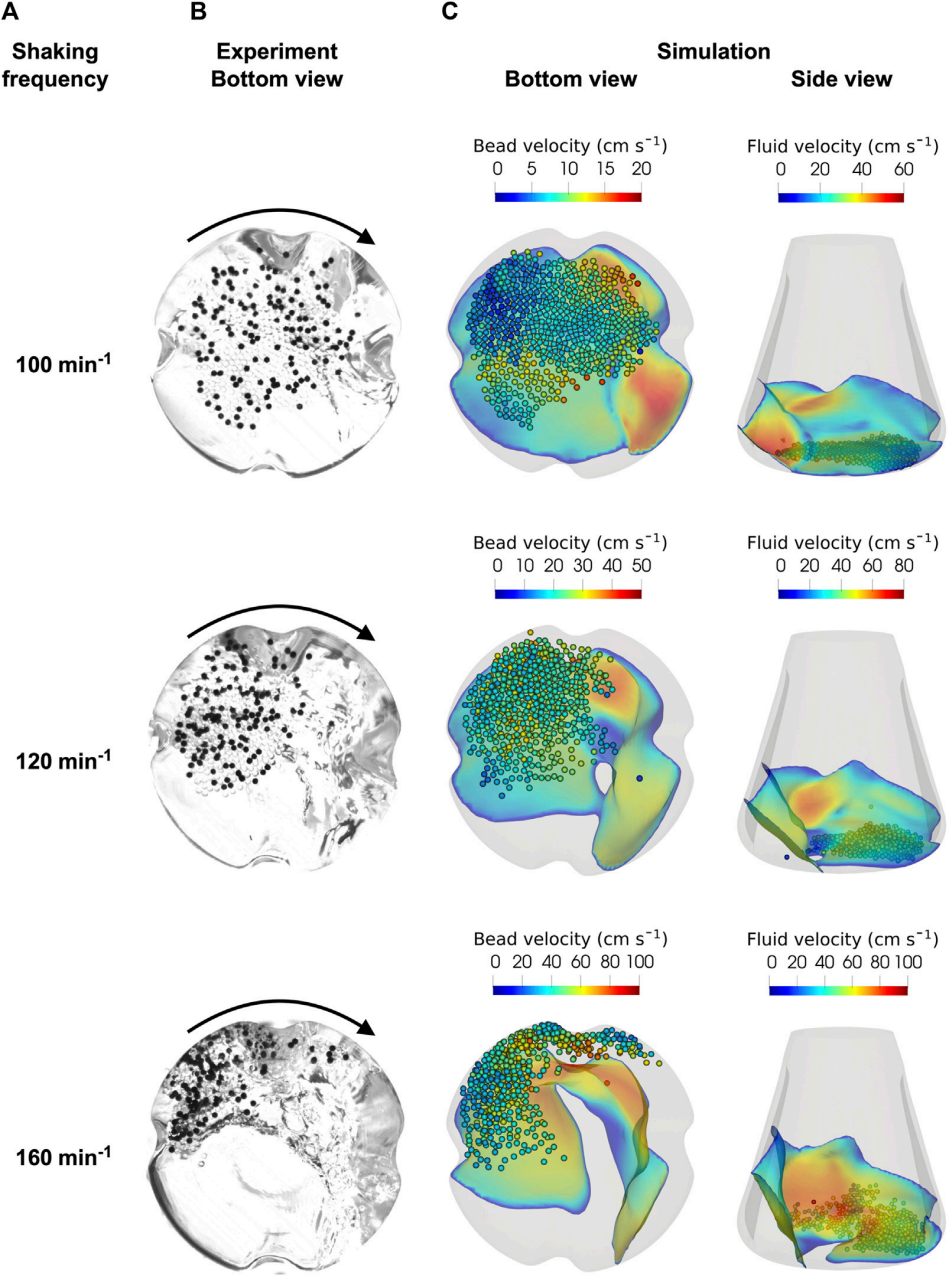
**(A)**: Glass beads (bead diameter 1.932 mm, bead volume concentration of 40 mL L^-1^) experimentally recorded at the bottom of the ShF at different shaker speeds. The black points are marker beads used in the PTV analysis; CFD-DEM simulations of the multiphase flow inside the ShF **(B)**: bottom view, **(C)**: side view.

In the study by Schrader et al. (2019), only the liquid level at the ShF was considered for the initial validation of the simulations. In the present study, experimental images of the beads at the bottom of the ShF were taken to verify the bead velocities calculated by the CFD-DEM simulations. Figure 8 shows the simulated and experimentally determined images for the largest glass bead diameter of almost 2 mm for three shaking frequencies between 100 and 160 min^-1^ used during the cultivations (note that corresponding videos to the images can be found in the Supplementary Materials). Next to the almost transparent glass beads are the black marker glass beads used to obtain the bead velocities using PTV. At the lowest shaking frequency of 100 min^−1^, the beads are distributed over a large area of the ShF bottom, but centrifugal forces pushed the beads further out as the speed increased. At a shaking frequency of 160 min^−1^, the beads were in a tightly packed bed in the curved part of the ShF bottom. This results in greater interaction between the baffles and the beads, further increasing the stresses. Moreover, the formation of multiple layers of beads made it difficult to detect of individual marker beads. Light reflections from the surface created dark artifacts that made image analysis even more complex. By using multiple light sources and a cardboard cylinder with aluminum foil surrounding the ShF, the light artifacts were reduced to a minimum.

The bottom and side views of the simulations show the glass beads and the fluid in the interface, colored according to their velocity. As in the experiments, the beads in the simulation are pushed further towards the outer diameter of the ShF as the shaking frequency increases. This significantly increases the velocity of both the beads and the liquid (note that the colors change with shaking frequency). In addition, the side view shows that the liquid is increasingly forced against the side walls. While at 100 min^−1^ there is almost exclusively a monolayer of beads on the bottom, at higher velocities, layers of beads are formed as already seen in the experiments. At a shaking frequency of 160 min^−1^, some beads are deflected at the baffles and moved upwards so that the beads are mixed more intensively. In summery, the simulations show a good qualitative agreement with the experiments. However, the beads are more densely packed in the experiments than in the simulations, especially at higher shaking frequencies. Nevertheless, the CFD-DEM simulations are able to describe the motion behavior of the beads in the ShF with sufficient accuracy.

#### 3.5.2 Bead velocity distributions from experiments and simulations

**FIGURE 9:**
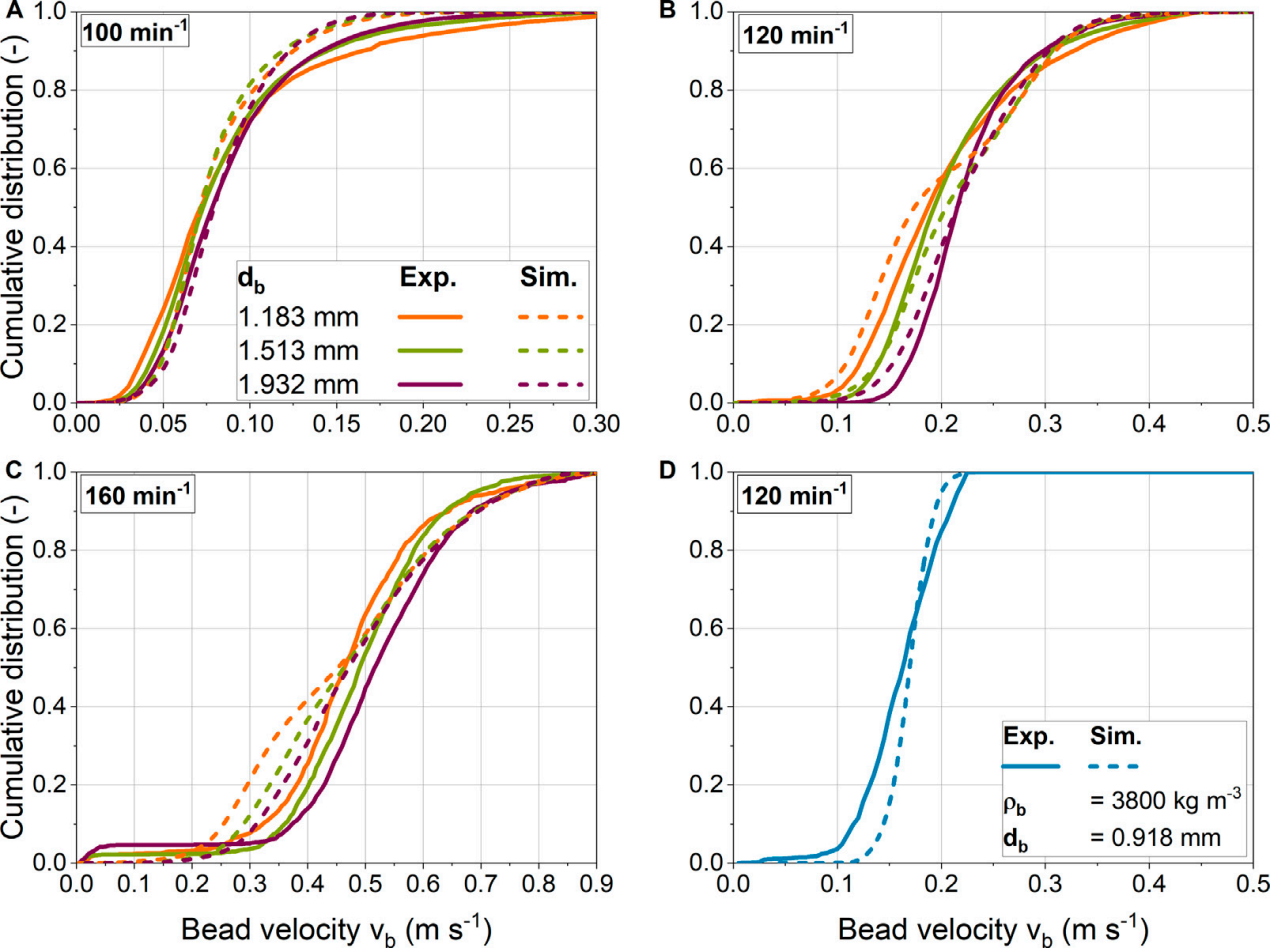
**(A-C)** Glass and **(D)** ceramic bead velocity distributions obtained from simulations and validation experiments for different shaking frequencies and bead diameters.

In addition the qualitative comparison in section 3.5.1, the velocities determined by PTV are compared with the results of simulations. Therefore, Figures 9A–C shows the cumulative bead velocities determined experimentally and numerically for three bead diameters and shaking frequencies. In general, analogous to the discussion in section 3.2.2, the bead velocity is higher than the shear velocity. This is because the overall bead velocity includes the translational rolling velocity in addition to the translational shear velocity. At the lowest shaking frequency of 100 min^−1^, the experimental distributions for different bead diameters are similar, indicating that bead size has little influence on bead velocity. At higher shaking frequencies, the distributions differ depending on bead size, especially in the lower velocity range. At the same time, the maximum velocities achieved are almost identical. A higher bead mass with larger bead diameters results in stronger centrifugal forces, so that more beads are transported to the edge of the ShF. Furthermore, a constant mass of beads was used, which means that the number of beads decreases with bead diameter to the power of three as the bead size increases. In addition, the area required per bead increases as the projection area increases with bead diameter to the power of two. Assuming a single layer of beads, the required area of all beads is the product of the number of beads and the projection area. Consequently, the area requirement increases inversely with decreasing bead diameter for a constant total bead mass concentration. This, in turn, results in more beads moving at lower velocity in the central region of the ShF for smaller bead sizes, and finally in a broader velocity distribution. In reality, smaller beads form vertical layers, which partially reduces the effect discussed before.

Overall, there is good agreement between the experimental and numerical distributions for the two lowest shaking frequencies of 100 and 120 min^−1^. However, the experimental distributions for 100 min^−1^ flatten out in the upper velocity range. It is noticeable that at a shaking frequency of 120 min^−1^, the two smallest bead sizes have a bimodal velocity distribution, while this effect is somewhat more pronounced for smaller beads. A likely cause is that the simulation data includes velocities from all vertical bead layers. In contrast, PTV captures only a two-dimensional velocity field without the frequency of bead velocities in the vertical direction. Accordingly, this method cannot capture the frequency of bead velocities in different bead layers. Smaller bead diameters tend to have more layers of beads, so the effect described above becomes more pronounced. Somewhat larger deviations between experiment and simulation are seen at the highest speed. The main reason for the deviations might be that the beads are now arranged very densely. This increases the likelihood of incorrect mappings of particle pairs between two images in the PTV analysis. Figure 8 shows that the beads at 160 min^−1^ are slightly more compressed in the experiment than in the simulation, so this could be another reason for the observed differences.

In addition to the glass beads, white ceramic beads were also investigated. The experimental and simulated mean and maximum velocities are close. However, the experimental distribution shows slightly lower velocities in the lower velocity range. Overall, it is shown that the presented experimental method is suitable to determine the velocities of the beads in the ShF. Moreover, the comparison between experiment and simulation shows a high degree of agreement. Accordingly, the chosen simulation approach is a suitable tool for the analysis and prediction of the stresses caused by the beads.

However, biomass was not considered in the simulations and experiments. The cultivation broth becomes turbid to non-transparent after a certain cultivation time. Thus, reliable identification and tracking of the beads on the camera images becomes much more difficult due to poorer illumination conditions. Depending on the morphological state of the culture and the biomass concentration, the viscosity of the culture broth increases and the viscosity behavior often becomes non-Newtonian (Olsvik and Kristiansen, 1994). The use of transparent model fluids may be a feasible approach to achieve realistic viscosity behavior while maintaining optical accessibility (Bliatsiou et al., 2020). It is expected that the general motion behavior of the beads is not significantly affected by collisions with pellets due to the higher inertia of the beads. However, biomass entrapment between the beads may alter (rolling) friction. This, in turn, would affect the intensity of bead stress.

### 3.6 Derivation of a characteristic parameter for the bead-induced stress

In the following chapter, novel model quantities for the bead-induced stress of microorganisms in ShFs are presented. The EDCF is often used to model fluid induced stress in aerated STBs on filamentous microorganisms. This concept shows that in addition to the intensity of stress, the active stress volume and SF are also decisive. However, the EDCF cannot be directly applied to the ShF due to the different types of power input and the associated stress mechanisms. In STB, filamentous microorganisms are subjected to turbulent stress due to the power input of the stirrer and aeration (Lin et al., 2010). CFD simulations of a STB showed that the normal Reynolds stresses were significantly greater than the shear Reynolds stresses (Eslahpazir Esfandabadi, 2013). The authors explained the observed reduction in pellet size by suggesting that the pellets were first deformed by normal forces and then damaged by erosion due to shear forces (Eslahpazir Esfandabadi, 2013). The beads in the ShF induce shear stress $S_{s}$ and normal stress $S_{n}$ in the contact zone. The shear rate (Eq. 7) in the contact point depends on the tangential shear velocity $v_{90,ts}$ of the beads and the size $d_{a}$ of the stressed bio-agglomerates.

$${\dot{\gamma }}_{90}=\frac{v_{90,ts}}{d_{a}}$$ (7)

$$S_{s}\propto v_{90,ts}$$ (8)

The size of the bio-agglomerates changes during the cultivation time, so only the shear velocity is considered for $S_{s}$ . For an agglomerate size of 400 μm, the maximum shear rate ranges from 100 to 1,000 s^−1^ depending on the shaking frequency. Thus, the shear rate is in the range of the average shear stress that occurs in the STB (Campesi et al., 2009). For smaller bioagglomerates, even significantly higher shear rates can occur. In addition to the shear rate, the normal stresses acting during shear also contribute to the damage of filamentous structures. Higher normal stresses are expected to compress the structures more, allowing shear forces to be more easily transmitted. For this reason, the gravitational force of a bead $F_{g}$ is used as a measure of the normal stresses.

$$S_{n}\propto F_{g}\propto m_{b}\cdot g\propto d_{b}^{3}\cdot \rho_{b}\cdot a_{g}\;\left(N\right)$$ (9)

In the EDCF, the stirrer swept volume serves as a measure of the size of the zone of sufficiently high stress. However, when beads are used, the number of beads $N_{c}$ in contact with the bottom (Eq. 10) correlates with the total shear stress area $A_{s}$ . The number of bead-wall contacts present in the shake flask at any given time was not directly available from the simulation data, so the SF for bead-wall contacts was assumed to be proportional to $N_{c}$ . It was also assumed that the projection area of a bead $A_{b}$ is proportional to the active shear area $A_{s}$ per bead.

$$A_{s}\propto N_{c}\cdot A_{b}\propto SF\cdot d_{b}^{2}\;\left(\frac{m^{2}}{s}\right)$$ (10)

$$SAR\propto \frac{S_{s}\cdot S_{n}}{A_{s}}=\frac{v_{90,ts}\cdot d_{b}^{3}\cdot \rho_{b}\cdot a_{g}}{SF\cdot d_{b}^{2}}=\frac{v_{90,ts}\cdot d_{b}\cdot \rho_{b}\cdot g}{SF}\left(\frac{N}{m}\right)$$ (11)

The combination of Eqs 8–10 results in a characteristic parameter called the stress area ratio (SAR) (Eq. 11), which describes the ratio of shear and normal stresses to the total active bead stress area. The product of normal and shear forces is based on the idea that sufficiently high normal forces must be present to effectively shear the cells in a pellet. Increasing normal forces results in greater compression of the mycelium and the build-up of tensile stresses in the mycelial structure. Additional shear can increase these stresses, resulting in the rupture of hyphae or even the destruction of entire pellets. The study by Rigali et al. (2008) hypothesized that N-acetylglucosamine released during cell wall hydrolysis triggers antibiotic formation in *Streptomyces*. Macroparticles could therefore potentially enhance the release of lysis substances due to mechanical forces and thereby increasing antibiotic formation. However, further research is needed to explain more precisely the relationship between bead addition and increased product formation.

### 3.7 Correlation of the stress area ratio with cultivation results

In previous studies (Walisko et al., 2017; Schrader et al., 2019; Schrinner et al., 2021), cultivations of the filamentous actinomycete *L. aerocolonigenes* were performed in ShFs with different process parameters. In these cultivations, the shaking frequency as well as the size, number and density of the beads were varied to investigate the influence on product formation. The addition of beads was found to have a decisive influence on the concentration of the antibiotic product rebeccamycin. The objective in this section was to correlate the rebeccamycin concentration and the average yield coefficient of different 10-day *L. aerocolonigenes* cultivations with previously presented simulation results to better understand the influence of bead stress.

**FIGURE 10:**
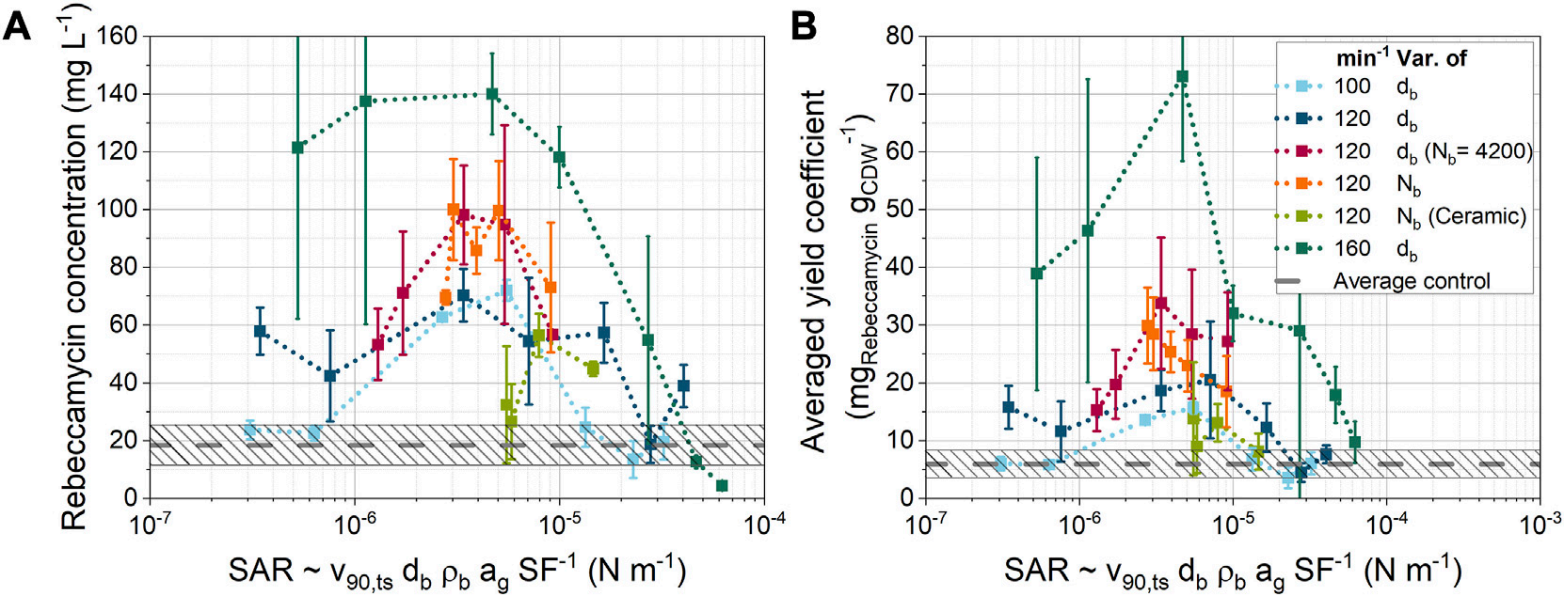
Dependency of **(A)** the rebeccamycin concentration and **(B)** the averaged yield coefficient [cultivation data from Schrader et al. (2019) and Schrinner et al. (2021)] on the SAR parameter derived from simulation data. Unless otherwise stated, only glass beads of different sizes and bead concentrations were used.

The Figure 10A shows the final product concentrations from 35 cultivations as a function of the model parameter SAR . It should be noted that each parameter series is an independent set of cultivations with different pre-cultures. It is often reported in the literature that cultivations of filamentous microorganisms from different pre-cultures can only be compared to a limited extent (Schrinner et al., 2020; Schrinner et al., 2021). Furthermore, high variability in the production of secondary metabolites in shake flask cultivation is a well-known challenge (Siebenberg et al., 2010; Sohoni et al., 2012). A comparison of *S. coelicolor* cultivations in shake flasks and deepwell microtiter plates showed a significantly lower variability (39% vs. 4%–9%) for the production of the antibiotic novobiocin in deep-well plates (Siebenberg et al., 2010). Transferring the simulation and modeling approach presented in this work to microtiter plates with beads may allow for a better understanding and modeling of bead-induced stress in the future.

Nevertheless, an optimum curve was found for the product concentration as a function of the newly defined model parameter. Only two data points for the ceramic ball series (light green) do not fall within the optimum curve. These points correspond to the two highest ceramic bead concentrations (19.7 and 26.3 mL L^−1^). It is also noticeable that the product concentrations are shifted on the *y*-axis with increasing shaking frequencies. This indicates that other effects related to shaking frequency may be affecting product formation.

Figure 10B shows the average yield coefficient, which is the ratio of the final product concentration to the final cell dry weight concentration after 10-day of cultivation. The overall trend is similar to that in Figure 10A, but a clearer trend is seen for the variation in the number of glass beads N_b_ at constant bead size. In this case, the average yield coefficient decreases with increasing SAR. An improved agreement with other cultivation parameters can also be observed for the variation of the number of ceramic beads. Thus, the changes in the number of beads seem to have a particular effect on the amount of biomass. Overall, product formation appears to be determined by the ratio of shear and normal forces to the dimension of the total shear stress area. These results show that compared to the mean value of all control cultivations without bead addition, the product concentration can be increased by a factor of about seven. In contrast, excessive stresses at the maximum shaking frequency of 160 min^−1^ led to lower product concentrations than the mean value of all control cultivations without beads (dashed line). Furthermore, these cultivations had lower bio dry mass concentrations than the corresponding unsupplemented control. Overall, the right amount of stress seems to be crucial for optimal cultivation results. In the literature, both positive and negative effects of bead addition have been reported for different strains and product types (Hotop et al., 1993; Dobson et al., 2008; Liang et al., 2008; Lee et al., 2010; Sohoni et al., 2012; Cai et al., 2014; Holtmann et al., 2017; Walisko et al., 2017; Song et al., 2018; Zhu et al., 2018; Schrader et al., 2019; Du et al., 2020; Jezkova et al., 2021; Králová et al., 2021; Schrinner et al., 2021; Li et al., 2022). It is therefore necessary to vary the stresses over a wide range in order to find the optimum cultivation conditions. For this purpose, the SAR can be used to compare different cultivation conditions. Careful pre-selection of cultivation parameters can avoid combinations with similar SARs, thus reducing the number of cultivations required to screen for the influence of mechanical stress.

## 4 Conclusion and outlook

Filamentous bacteria and fungi are particularly relevant for biotechnological production processes due to their broad product spectrum. In order to achieve high product formation, an optimal cellular morphology must be set during cultivation, depending on the strain and the product formation kinetics. Thereby, the cellular morphology can range from dispersed mycelium to clumps to compact pellet structures. For this reason, it is crucial to determine the optimal cultivation conditions as early as possible in the development of a biotechnological process. At the beginning of the scale-up process, ShFs are widely used for cultivation due to their ability to parallelize. The turbulent stresses in ShFs are significantly lower than in STBs, so several studies have added macroparticles to the medium to increase the stress. In detail, bead size, number of beads, bead density, and shaking frequency have been varied, resulting in either a positive or negative effect on the cultivation process. While the EDFC concept for describing mechanical stresses exists for the STB scale, a model approach for the use of macroparticles in ShFs is lacking.

Therefore, as a first step in this direction, an existing setup for CFD-DEM simulations of a ShF was used in this study. The analysis of the bead contact types showed that the translational shear between the beads and the bottom of the ShF is the largest. Next, different combinations of bead size, number of beads, bead density and shaking frequency were systematically varied to investigate the dependence of bead-wall shear velocity and bead-wall SF on these parameters.

Increasing the volume concentration of the beads resulted in an increase in the maximum shear velocity between the wall and the beads up to a certain filling level. Thereafter, the shear velocity stagnated or decreased as the bottom of the ShF became highly covered by the beads. A higher number of beads resulted in the formation of bead layers due to the limited space at the bottom of the flask, creating new shear zones between the layers. Increasing the shaking frequency increased the shear at the bottom and promoted the formation of bead layers at the edge of the flask. The bead size affected the shear velocity differently depending on the shaking frequency. Moreover, the SF between the beads and the shaking flask bottom depended on the shaking frequency, bead size, and bead concentration.

In order to compare the bead induced stresses with the results of filamentous and shear sensitive *Lentzea aerocolonigens* cultivations, a new characteristic parameter was defined. This characteristic parameter, named stress area ratio (SAR), takes into account the ratio of bead-wall shear and normal stresses to the total shear area. Comparison of the SAR with cultivation results showed an optimum pattern for both product concentration and mean product-to-biomass related yield coefficient. Accordingly, the model is able to compare different macroparticle-enhanced cultivation conditions.

To validate the simulation method, the beads on the bottom of the ShF were recorded using a high-speed camera. The images were used to determine the velocities of the beads using particle tracking velocimetry. The comparison with the simulations showed a high agreement between simulation and experiment. Thus, the chosen simulation approach is a powerful method for quantifying bead-induced stresses in ShFs.

In the future, the applicability of the model to other shear sensitive (filamentous) strains should be verified. Furthermore, more investigations at high shaking frequencies are necessary to explain the strongly increased product concentrations. Overall, this study will help to more systematically find the optimal cultivation conditions for shear sensitive microorganisms at the ShF scale. For a successful model-based scale-up to a STB, further research is needed to establish a relationship between the bead and fluid induced stresses. In addition, the simulations provide data for a future simulation-based investigation of the consequences of mechanical shear and normal stress at the level of bio-pellet.

## Figures and Tables

**Table:** 

**Notation**
***a_c_***	Centrifugal acceleration $\left(m\;s^{-2}\right)$
$A_{b}$	Bead cross-sectional area $\left({cm}^{2}\right)$
$A_{s}$	Stress area $\left(m^{2}\right)$
**CFD**	Computational fluid dynamics
**COR**	Coefficient of restitution $\left(-\right)$
**c**	Coupling interval $\left(-\right)$
$c_{v,b}$	Bead volume concentrations $\left(mL\;L^{-1}\right)$
$d_{a}$	Bioagglomerate diameter $\left(m\right)$
$d_{b}$	Bead diameter $\left(mm\right)$
$d_{s}$	Stirrer diameter $\left(m\right)$
**DEM**	Discrete element method
**EDCF**	Energy dissipation circulation function $\left(W\;m^{-3}\;s^{-1}\right)$
f	Shake frequency $\left(s^{-1}\right)$
$F_{\mathrm{ac}}$	Centrifugal force $\left(N\right)$
$F_{g}$	Gravity force $\left(N\right)$
**g**	Gravitational acceleration $\left(m\;s^{-2}\right)$
$m_{b}$	Bead mass $\left(kg\right)$
$N_{b}$	Number of beads $\left(-\right)$
$N_{c}$	Number of contacts $\left(-\right)$
$p_{f}$	Fracture probability $\left(-\right)$
$P_{V,\max}$	Maximum local volumetric power consumption $\left(W\;m^{-3}\right)$
$P_{v}$	Mean volumetric power consumption $\left(W\;m^{-3}\right)$
**PTV**	Particle tracking velocimetry
$r_{orb}$	Orbital radius of the shaker $\left(m\right)$
$r_{rot}$	Rotation radius $\left(m\right)$
$S_{s}$	Bead induced shear stress $\left(-\right)$
$S_{n}$	Bead induced normal stress $\left(-\right)$
SAR	Stress area ratio $\left(kg\;s^{-2}\right)$
**SE**	Stress energy $\left(J\right)$
**SF**	Stress frequency $\left(s^{-1}\right)$
**ShF**	Shake flask
**STB**	Stirred tank bioreactor
$t_{c}$	Circulation time $\left(s\right)$
∆t	Time step width $\left(s\right)$
$v_{b}$	Bead velocity $\left(m\;s^{-1}\right)$
$v_{90,ts}$	Shear velocity $\left(m\;s^{-1}\right)$
$Y_{b}$	Young’s modulus $\left(Pa\right)$
**Greek letters**	
${\dot{\gamma }}_{b}$	Bead shear rate $\left(s^{-1}\right)$
ϵ	Turbulent energy dissipation rate $\left(m^{2}\;s^{-3}\right)$
$\eta_{m}$	Medium dynamic viscosity $\left(mP\;s\right)$
$\eta_{w}$	Water dynamic viscosity $\left(mP\;s\right)$
$\theta_{m}$	Medium contact angle (°)
$\theta_{w}$	Water contact angle (°)
µ	Friction coefficient (°)
$\mu_{r}$	Rolling friction coefficient (−)
$\nu_{b}$	Poisson’s ratio (−)
$\rho_{b}$	Bead density $\left(kg\;m^{-3}\right)$
$\rho_{m}$	Medium density $\left(kg\;m^{-3}\right)$
$\rho_{w}$	Water dynamic viscosity $\left(kg\;m^{-3}\right)$
$\sigma_{m}$	Medium surface tension $\left(mN\;m^{-1}\right)$
$\sigma_{w}$	Water surface tension $\left(mN\;m^{-1}\right)$

## Data Availability

The raw data supporting the conclusion of this article will be made available by the authors, without undue reservation.
