# Supplementary material for: Quantification and modeling of macroparticle-induced mechanical stress for varying shake flask cultivation conditions
Source: Front Bioeng Biotechnol. 2023 Sep 4;11:1254136. doi: 10.3389/fbioe.2023.1254136 (PMC10507416; doi:10.3389/fbioe.2023.1254136)
Supplement: Supplementary file 12 [file DataSheet1.docx]

Supplementary Material

Quantification and modeling of macroparticle-induced mechanical stress for varying shake flask cultivation conditions

Marcel Schrader^*^; Kathrin Schrinner; Laura Polomsky; Dimitri Ivanov; Carsten Schilde; Ingo Kampen; Rainer Krull; Arno Kwade

*** Correspondence:** Marcel Schrader: marcel.schrader@tu-braunschweig.de

# Supplementary Figures and Videos


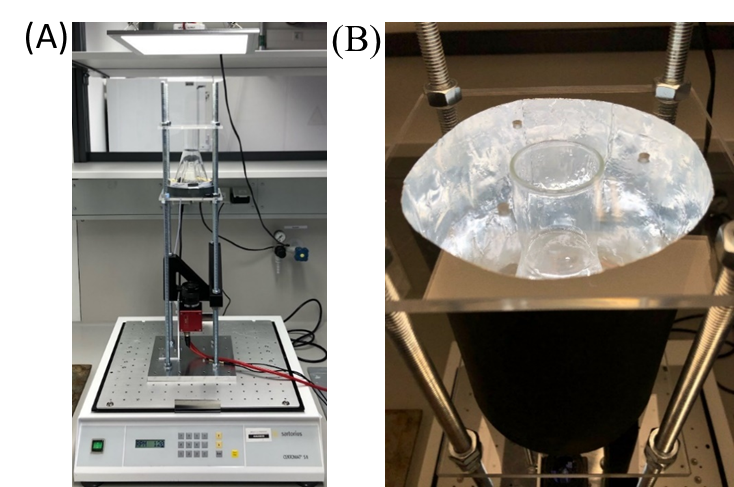


**S1** **(A):** Experimental setup for recording the bead movement at the bottom of the shake flask using a high-speed camera. The ropes used to stabilize the setup at high shaking frequencies and the cardboard cylinder are missing; **(B):** Close-up of the shake flask with the cardboard cylinder covered on the inside with aluminum foil.

**S2** **(A)** Ratio of the bead-wall (BW) and bead‑bead (BB) shear velocities for different bead diameters and shaking frequencies; **(B)** Ratio of the bead-wall (BW) and bead‑bead (BB) shear velocities for different bead volume concentrations.


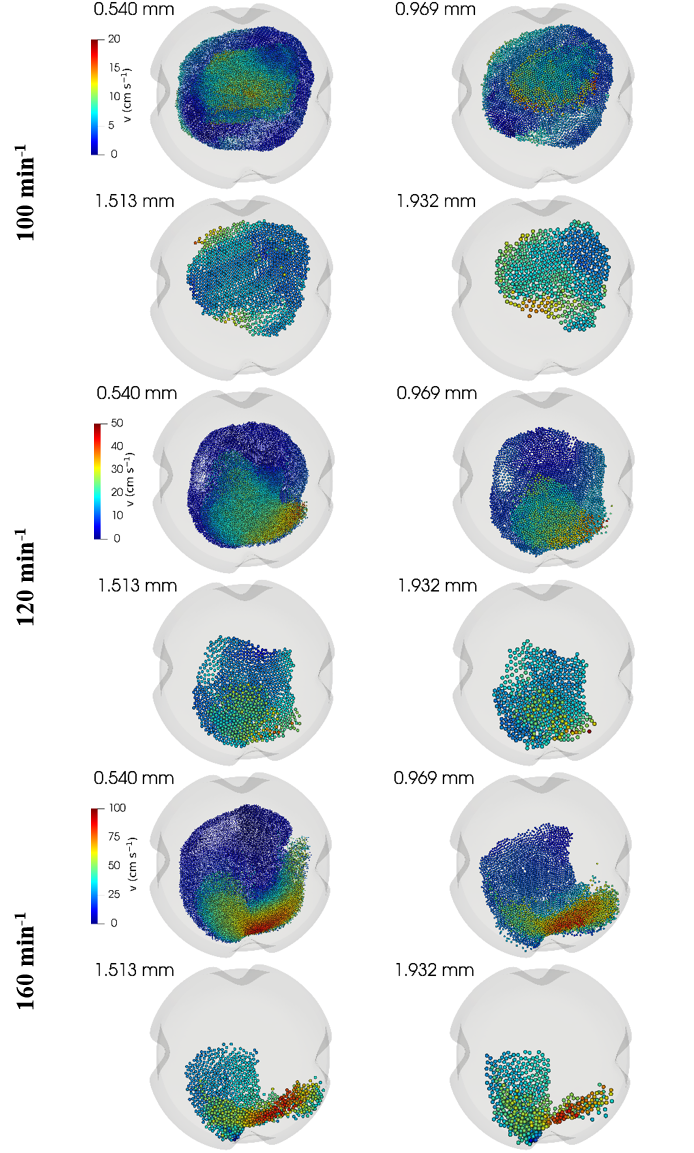


S3 Top view on the shake flask with different glass bead diameters and shaking frequencies at a constant bead volume concentration of 40 mL L^-1^. Note: In addition to the images, the corresponding videos (V1-V3) can be found in the additional materials.


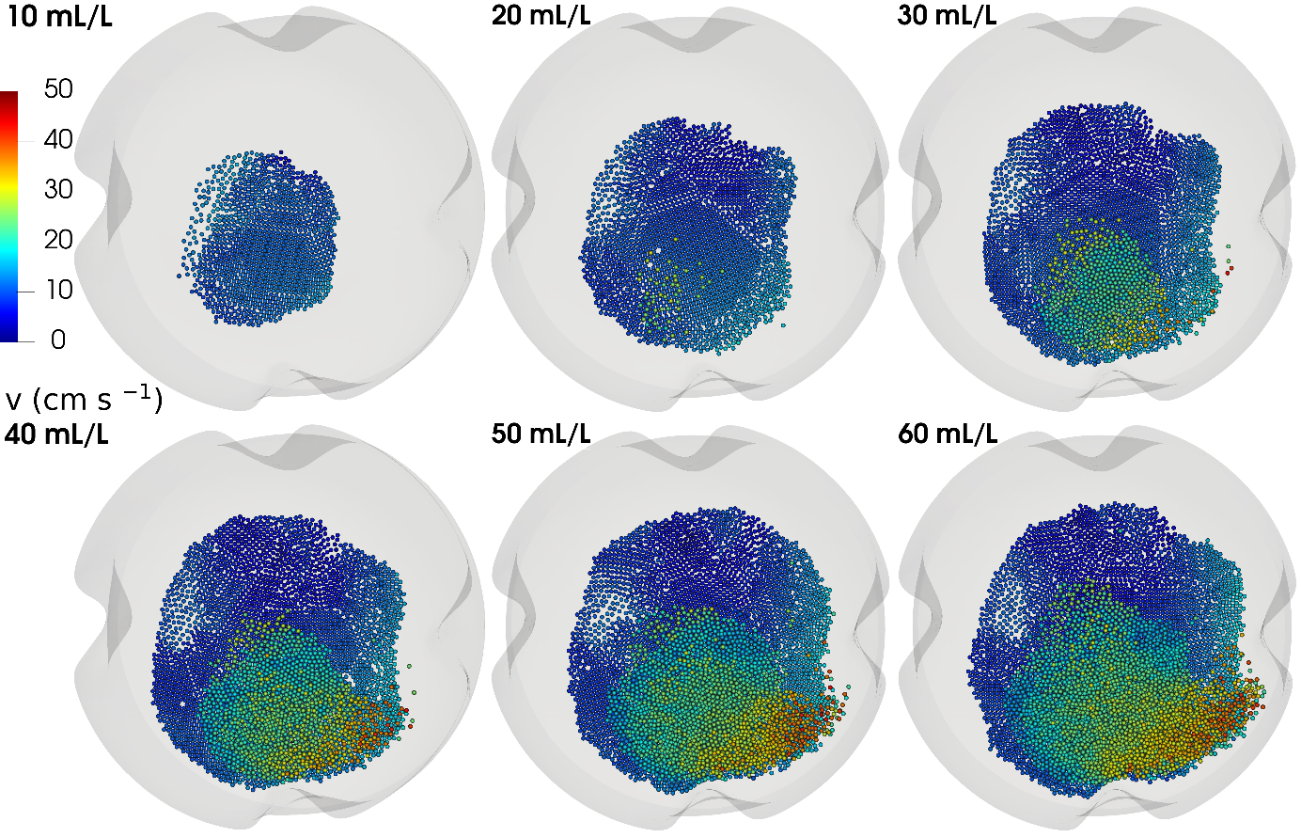


S4 Variation of glass bead volume concentration (top view) at 120 min^-1^. Note: In addition to the images, the corresponding video V4 can be found in the additional materials.


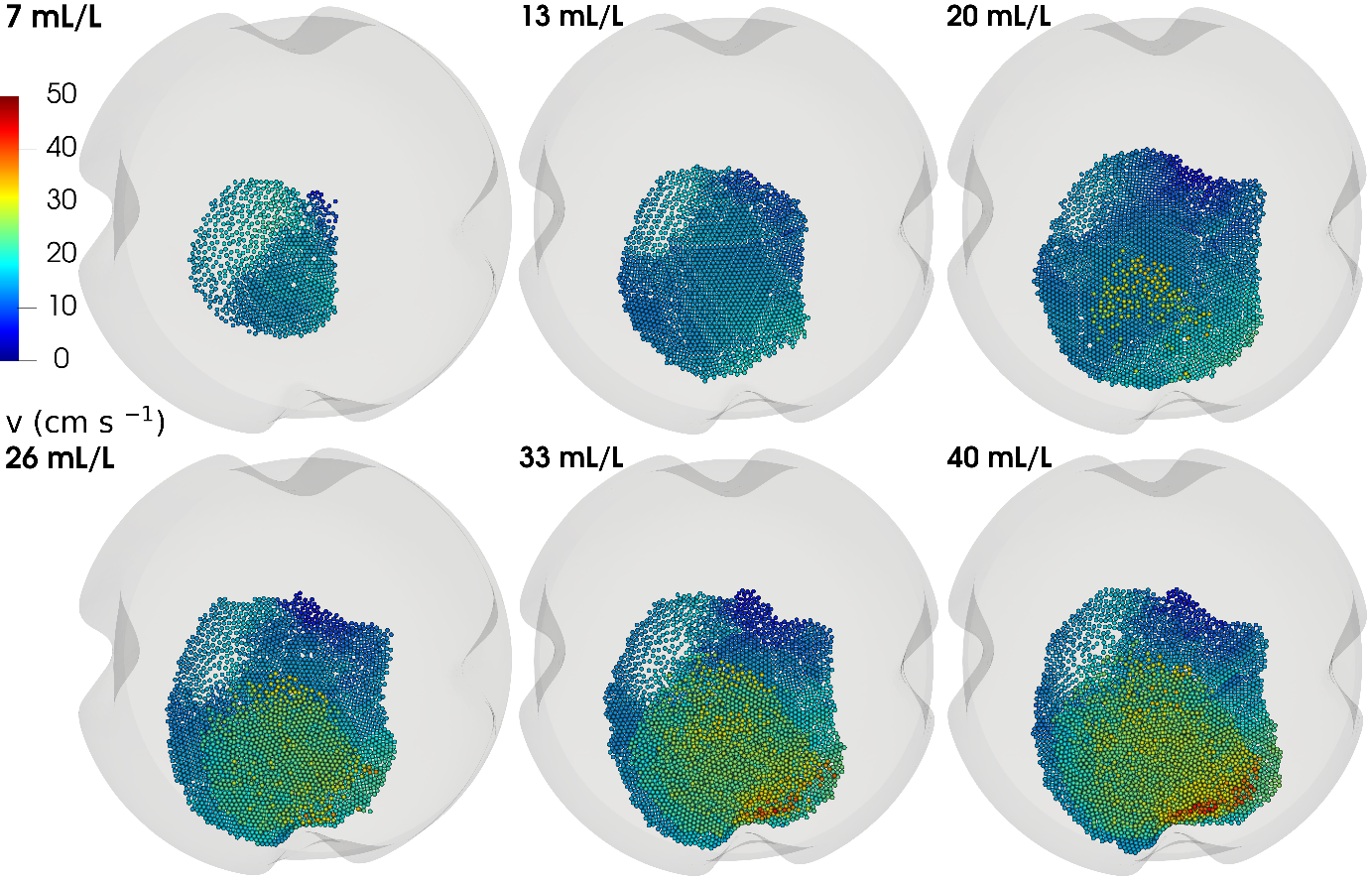


S5 Variation of ceramic bead volume concentration (top view) at 120 min^-1^. Note: In addition to the images, the corresponding video V5 can be found in the additional materials.


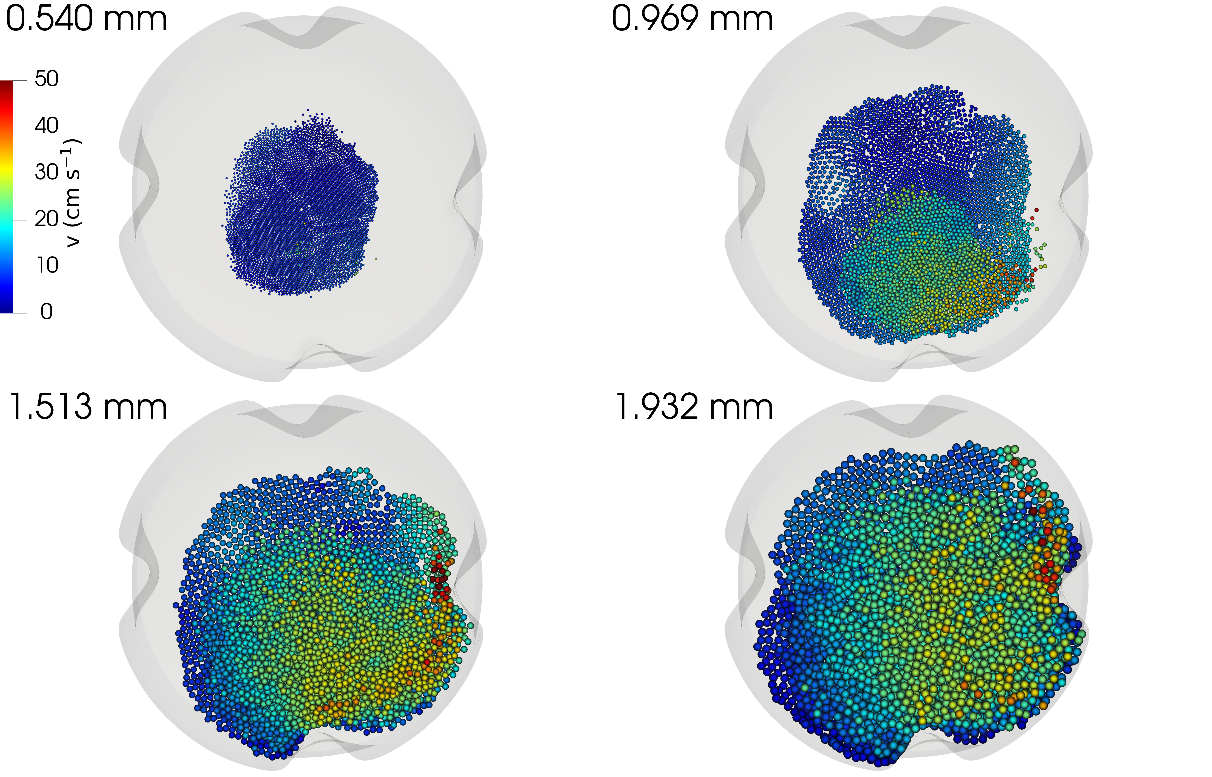


S6 Variation of glass bead diameter (top view) at 120 min^-1^ with a constant number of beads of 4200. Note: In addition to the images, the corresponding video V6 can be found in the additional materials.

**S7** Overview of a part of the videos, which were used for validation.

| **Video number** | **Shaking frequency** | **Bead diameter** | **Bead volume concentration** |
| --- | --- | --- | --- |
| (experiment/ simulation) | $f$ | $d_{b}$ | $c_{v, b}$ |
|  | (min^-1^) | (mm) | (mL L^-1^) |
| **V7/ V8** | 100 | 1.932 | 40.0 |
| **V9/ V10** | 120 | 1.932 | 40.0 |
| **V11/ V12** | 160 | 1.932 | 40.0 |
